# Correspondence between Monkey Visual Cortices and Layers of a Saliency Map Model Based on a Deep Convolutional Neural Network for Representations of Natural Images

**DOI:** 10.1523/ENEURO.0200-20.2020

**Published:** 2021-01-12

**Authors:** Nobuhiko Wagatsuma, Akinori Hidaka, Hiroshi Tamura

**Affiliations:** 1 Faculty of Science, Toho University, Miyama 2-2-1, Funabashi, Chiba 274-8510; 2 School of Science and Engineering, Tokyo Denki University, Ishizaka, Hatoyama-machi, Hiki-gun, Saitama 350-0394; 3 Graduate School of Frontier Biosciences, Osaka University, 1-4 Yamadaoka, Suita, Osaka 565-0871; 4 Center for Information and Neural Networks (CiNet), 1-4 Yamadaoka, Suita, Osaka 565-0871

**Keywords:** attention, computational model, deep learning, saliency map, V1 saliency hypothesis, visual system

## Abstract

Attentional selection is a function that allocates the brain’s computational resources to the most important part of a visual scene at a specific moment. Saliency map models have been proposed as computational models to predict attentional selection within a spatial location. Recent saliency map models based on deep convolutional neural networks (DCNNs) exhibit the highest performance for predicting the location of attentional selection and human gaze, which reflect overt attention. Trained DCNNs potentially provide insight into the perceptual mechanisms of biological visual systems. However, the relationship between artificial and neural representations used for determining attentional selection and gaze location remains unknown. To understand the mechanism underlying saliency map models based on DCNNs and the neural system of attentional selection, we investigated the correspondence between layers of a DCNN saliency map model and monkey visual areas for natural image representations. We compared the characteristics of the responses in each layer of the model with those of the neural representation in the primary visual (V1), intermediate visual (V4), and inferior temporal (IT) cortices. Regardless of the DCNN layer level, the characteristics of the responses were consistent with that of the neural representation in V1. We found marked peaks of correspondence between V1 and the early level and higher-intermediate-level layers of the model. These results provide insight into the mechanism of the trained DCNN saliency map model and suggest that the neural representations in V1 play an important role in computing the saliency that mediates attentional selection, which supports the V1 saliency hypothesis.

## Significance Statement

Trained deep convolutional neural networks (DCNNs) potentially provide insight into the perceptual mechanisms of biological visual systems. However, the relationship between artificial and neural representations for determining attentional selection and gaze location has not been identified. We compared the characteristics of the responses in each layer of a DCNN model for predicting attentional selection with those of the neural representation in visual cortices. We found that the characteristics of the responses in the trained DCNN model for attentional selection were consistent with that of the representation in the primary visual cortex (V1), suggesting that the activities in V1 underlie the neural representations of saliency in the visual field to exogenously guide attentional selection. This study supports the V1 saliency hypothesis.

## Introduction

Attentional selection enables the brain to allocate its computational resources to the most important part of a visual scene at a specific moment (Posner, 1980) and establish visual perception (Carrasco, 2011; Yang et al., 2018). Visual saliency mediates attentional selection and underlies the determination of gaze location (Koch and Ullman, 1985; Zhang et al., 2012). Saliency maps have been proposed as a biologically plausible model for predicting attentional selection within the presented visual scene (Itti and Koch, 2000). In this model, the most salient location in a visual scene induces attentional selection. From the original model (Itti et al., 1998), various saliency map models based on the visual system have been proposed (Russell et al., 2014; Wagatsuma, 2019; Uejima et al., 2020) in which the activities of model neurons in early vision are the first, and necessary, process for organizing the saliency map. The crucial role of responses in the primary visual cortex (V1) used for computing visual saliency has been demonstrated by various studies, including physiological, psychophysical, and computational works (V1 saliency hypothesis; Li, 1999a, 2002; Jingling and Zhaoping, 2008; Zhaoping, 2014, 2019; Yan et al., 2018).

The deep neural network approach can be used to delve even more deeply into understanding the mechanism of sensory cortical processing (Yamins and DiCarlo, 2016). Deep convolutional neural network (DCNN) models, such as AlexNet (Krizhevsky et al., 2012), significantly improve object recognition for computer vision and provide a rich interconnection between neuroscientific and artificial approaches to explain the mechanism of visual systems (Yamins and DiCarlo, 2016; Geirhos et al., 2018). After AlexNet was trained on a large-scale dataset, model neurons in its early layers demonstrated selectivity to orientation and spatial frequency (Zeiler and Fergus, 2013), similar to V1 neurons (Hubel and Wiesel, 1968) and Gabor filters (Deco and Lee, 2004; Sakai et al., 2012). Pospisil et al. (2018) reported that many model neurons in AlexNet selectively respond to object boundaries and their curvature, which is similar to the neuronal characteristics of the intermediate visual area (V4; Pasupathy and Connor, 2001). These studies demonstrated that the mechanisms used by DCNN models for object recognition correspond, at least in part, to the hierarchical structure of the ventral visual stream for object perception (Le et al., 2012; Mahendran and Vedaldi, 2014).

**Figure 1.**
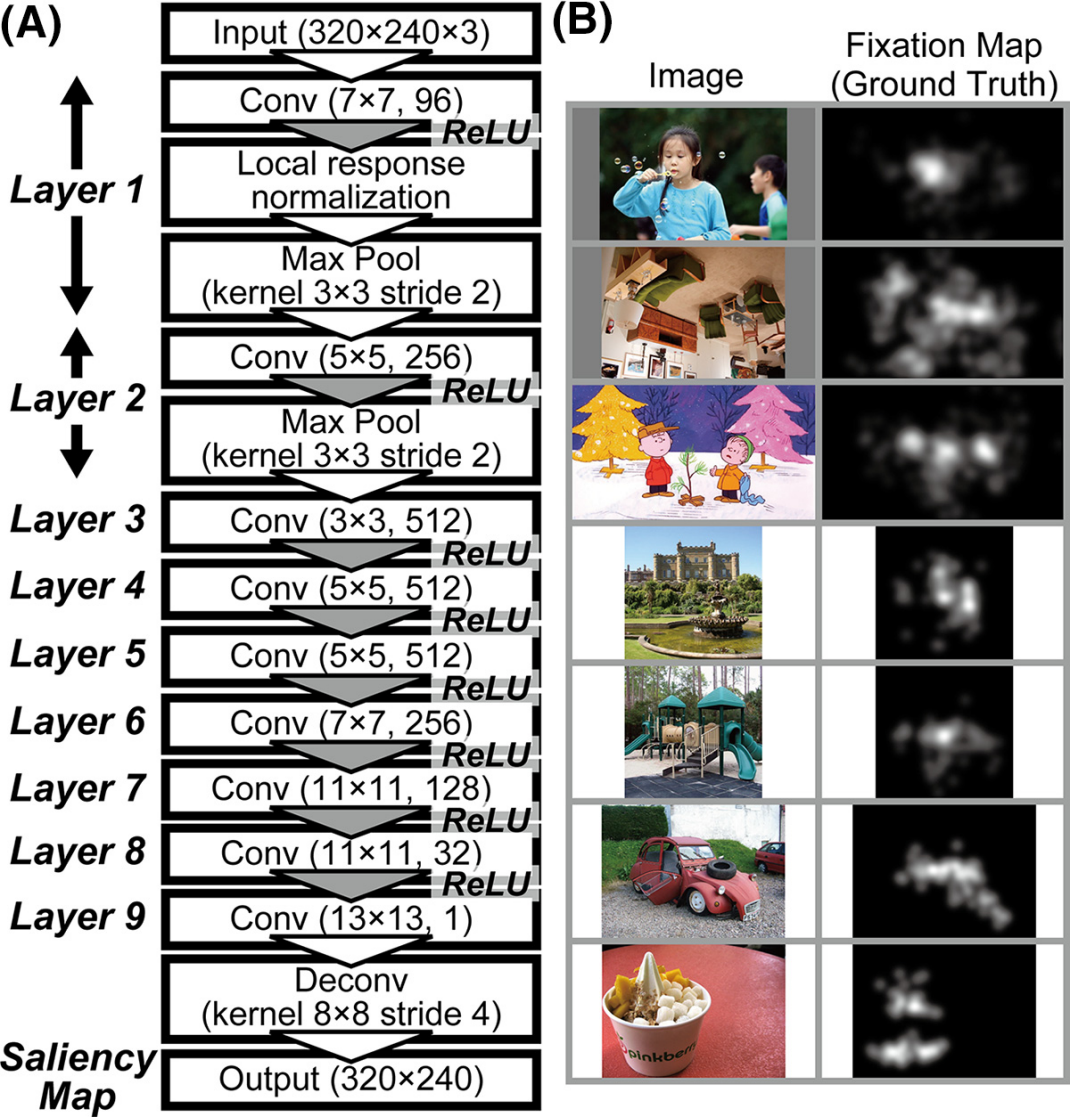
***A***, CNN architecture of the saliency map model proposed by Pan et al. (2016). This DCNN consists of nine convolutional (Conv), two max pooling (Max Pool), and one deconvolutional (Deconv) layers. We applied a variety of natural images with an intensity value ranging from 0 to 1 (RGB color images, 320 × 240 pixels) to the input layer of the network. Human fixation data were provided to the network as the ground truth images for learning the characteristics of human fixation locations. ***B***, Examples of the natural images and associated eye-fixation maps (ground truth) that were used to train the DCNN (Pan et al., 2016) for the generation of saliency maps. In total, 11,580 natural images and human fixation data were applied to the DCNN to generate the saliency map. The eye-fixation maps were generated by combining the fixation points across all images, followed by the convolution of the combined points with a 2D Gaussian function (for details, see Judd et al., 2009).

Deep neural networks have been used as a powerful modern tool to achieve and develop advanced saliency map models. Pan et al. (2016) proposed a saliency map model based on a DCNN (Fig. 1*A*), which outperformed previous models based on the visual system for the prediction of human gaze location. However, the mechanism underlying the DCNN saliency map model after it is trained remains unknown. Additionally, the relationship between artificial and neural representations of attentional selection for gaze location has not been elucidated at the layer level. Analyses of the DCNN saliency map model will provide crucial insight into the role of V1 responses underlying attentional selection.

To understand the interconnections between the mechanisms of DCNN saliency map models and neural systems for determining attentional selection, we investigated the correspondence between a DCNN saliency map model and monkey visual cortices for natural image representations. We trained the DCNN proposed by Pan et al. (2016) in saliency map generation based on natural image input (Fig. 1*B*) using various saliency datasets, including natural images and associated eye-fixation data. We quantitatively compared the characteristics of model neurons in each layer of the DCNN saliency map model with those of the neural representations in V1, V4, and the inferior temporal cortex (IT). Regardless of the DCNN layer level, the characteristics of the responses in the DCNN saliency map model were consistent with that of the neural representation in V1. We found marked peaks of correspondence between V1 and early level and higher-intermediate-level layers. These results suggest that the neural representation in V1 has a crucial role in computing saliency that underlies attentional selection and that mediates the determination of gaze location, which supports the V1 saliency hypothesis.

## Materials and Methods

### Physiologic experiments and responses of monkey visual cortices to natural object surfaces

**Figure 2.**
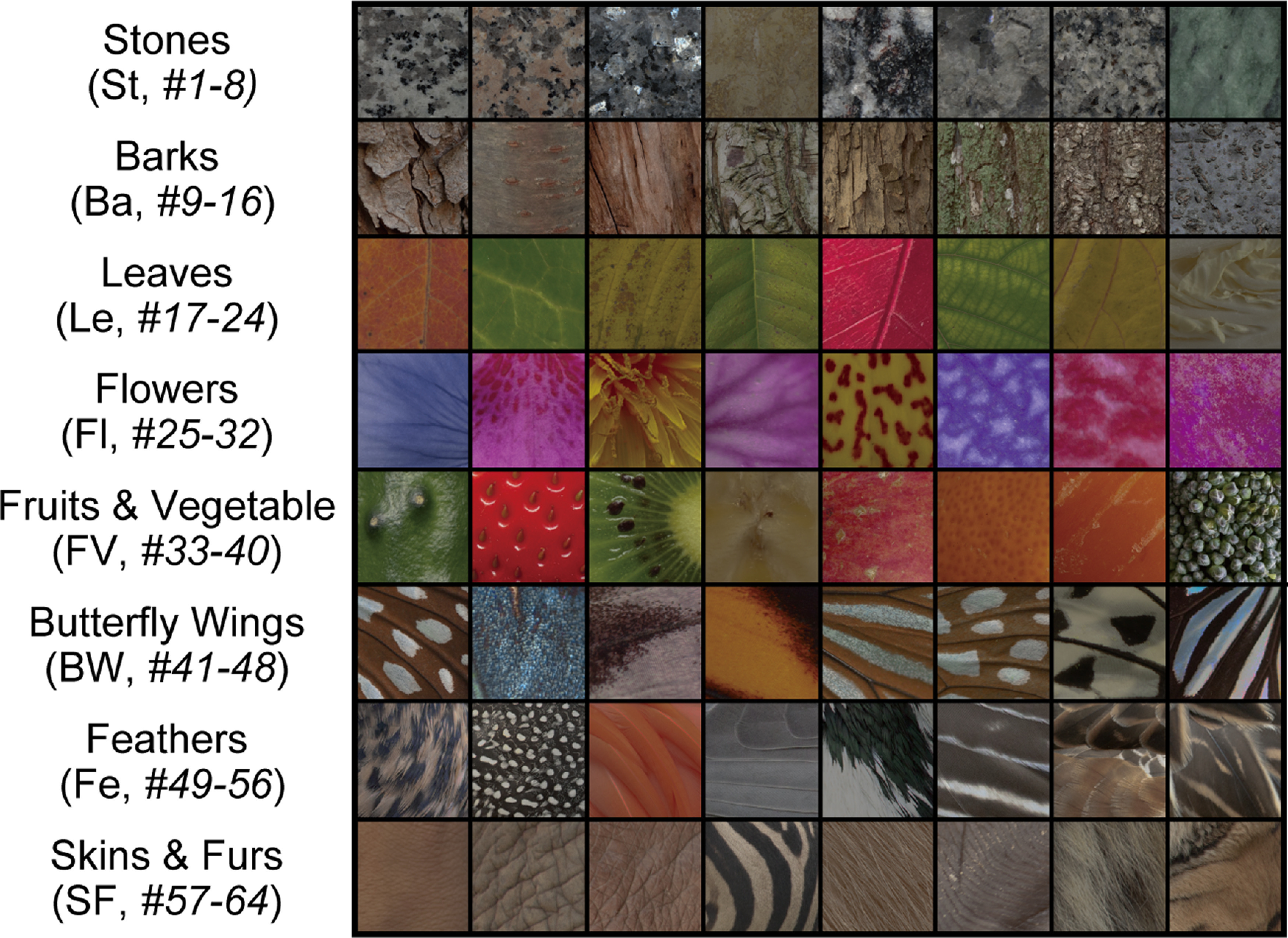
Stimuli consisting of natural object surfaces used for recording neuronal responses in V1, V4, and IT from *M. fuscata* by Tamura et al. (2016). A stimulus set in that physiological study consisted of 64 images of eight types of natural objects: stones (St; *n* = 8, #1–8), tree barks (Ba; *n* = 8, #9–16), leaves (Le; *n* = 8, #17–24), flowers (Fl; *n* = 8, #25–32), fruits and vegetables (FV; *n* = 8, #33–40), butterfly wings (BW; *n* = 8, #41–48), feathers (Fe; *n* = 8, #49–56), and skins and furs (SF; *n* = 8, #57–64). In that study, the responses of V1, V4, and IT neurons to these images were recorded to investigate how the surface visual features derived from natural objects are presented in these visual cortices. Additionally, these images were provided to the DCNN saliency map model (Pan et al., 2016) to analyze the characteristics of the responses of model neurons.

In the present study, we analyzed data obtained in a previous study conducted by Tamura et al. (2016). In that study, Tamura and colleagues extracellularly recorded the responses of a single neuron in the V1, V4, and IT of four monkeys (*Macaca fuscata*; two males and two females, body weight 5.9–8.6 kg) to images of natural object surfaces (Fig. 2) to investigate how surface-related features derived from natural objects are represented in the visual cortical areas. All experiments were performed in accordance with the guidelines of the National Institutes of Health (1996) and Japan Neuroscience Society and approved by the Osaka University Animal Experiment Committee.

The experimental procedures were similar to those of their previous study (Tamura et al., 2014). The monkeys were prepared during aseptic surgery, in which a head restraint was implanted. Additionally, the lateral and occipital part of the skull over the recording region was covered with acrylic resin. These surgical procedures were performed under full anesthesia via inhalation of 1–3% isoflurane (Forane, Abbott Japan) in nitrous oxide (70% N_2_O, 30% O_2_) through an intratracheal cannula. The monkeys were given an antibiotic (Pentcillin, Toyama Chemical; 40 mg/kg, i.m.), and an anti-inflammatory and analgesic agent (Voltaren, Novartis; or Ketoprofen, Nissin Pharmaceutical) immediately after surgery. The administration of the antibiotic, and anti-inflammatory and analgesic agent was maintained during the first postoperative week. After one to two weeks of recovery, the monkeys’ eyes were examined to enable the selection of appropriate contact lenses that allowed images placed 57 cm from the cornea to be focused on the retina. Photographs of the retinal fundus were used to determine the position of the fovea.

On the day of neural recording, the monkeys were sedated using intramuscular injections of atropine sulfate (0.1 mg/kg) and ketamine hydrochloride (12 mg/kg). During the preparation for neural recording, the monkeys were analgesized via inhalation of 1–3% isoflurane in nitrous oxide (70% N_2_O, 30% O_2_) through an intratracheal cannula. These were infused with the opioid fentanyl citrate (Fentanest, Daiichi Sanyo; 0.035 mg/kg/h) in lactated Ringer’s solution. Tamura and colleagues drilled a small hole (∼5 mm) in the resin-covered skull and made a small slit (2 mm) in the dura. They inserted an electrode through the slit to enable the recording of the neuronal responses.

Tamura and colleagues dilated the pupil of the eye contralateral to the recording hemisphere and relaxed the lens of the eye using 0.5% tropicamide/0.5% phenylephrine hydrochloride (Mydrin-P, Santen). They then covered the cornea of the eye with a contact lens of appropriate refractive power and curvature, and an artificial pupil (diameter, 3 mm) so that the eye would focus on images placed 57 cm away. After the electrode for recording the neuronal responses was inserted, they added vecuronium bromide (Masculax, MSD; 0.06 mg/kg/h) to the infusion solution to prevent eye movement during recording. Thus, the monkeys passively viewed stimuli on the display without eye movement.

Tamura and colleagues made single-unit recordings from V1, V4, and IT using a single-shaft electrode with 32 recording probes arranged linearly (A1X32-10 mm 50–413, A1X32-10 mm 100–413; NeuroNexus) or an eight-shaft electrode, where each shaft was a tetrode with four recording probes at the tip arranged in a rhombus (A8X1 tetrode-2 mm 200–312; NeuroNexus), and the centers of adjacent shafts were 0.2 mm apart. The distance between the centers of adjacent recording probes was 50 or 100 μm when using the single-shaft electrode and 25 μm when using the eight-shaft electrode. The activity of a single neuron was isolated offline using custom-made software to avoid the problem caused by spiking activity from the same neuron being recorded by two or more adjacent probes (for details, see Kaneko et al., 1999, 2007; Tamura et al., 2014). The recording sites in V1 were located on the surface of the occipital cortex, well behind the lunate sulcus. Those in V4 were located between the superior temporal sulcus and the lunate sulcus. Those in the IT cortex were located between the superior temporal sulcus and the anterior middle temporal sulcus, and anterior to the posterior middle temporal sulcus. After each recording session, the monkeys received analgesics and antibiotics. Each recording session lasted up to 7 h, and the monkeys had at least a week’s rest between recording sessions.

The stimulus set used by Tamura et al. (2016) consisted of 64 images of eight types of natural objects (Fig. 2): stones (St; *n* = 8, #1–8), tree barks (Ba; *n* = 8, #9–16), leaves (Le; *n* = 8, #17–24), flowers (Fl; *n* = 8, #25–32), fruits and vegetables (FV; *n* = 8, #33–40), butterfly wings (BW; *n* = 8, #41–48), feathers (Fe; *n* = 8, #49–56), and skins and furs (SF; *n* = 8, #57–64). The stimuli (6° × 6° in visual angle) were displayed on a liquid crystal display monitor (CG275W, Eizo) that was calibrated via an internal calibrator and checked using a spectrometer (Minolta CS-1000). The luminance values of the white and black areas were 125 and 1.3 cd/m^2^, respectively. Each stimulus was presented once monocularly for 200 ms against a homogeneous gray background to the eye contralateral to the recording hemisphere, and a homogeneous gray blank screen was presented for intervals of 200 ms between each presentation. This stimulus-presentation procedure was repeated for 25 or 30 blocks during each recording session, with the stimulus order pseudorandomized in each block.

The magnitude of a visually evoked response to a given stimulus was computed based on the firing rate recorded during the 200-ms stimulus-presentation period. To compensate for response latency, the beginning of the 200-ms window of stimulus presentation was shifted to 80 ms after stimulus onset for V1, V4, and IT neurons. The responsiveness of each neuron was qualitatively evaluated by comparing the firing rates recorded during the stimulus-presentation period across stimuli (Kruskal–Wallis test, *p *<* *0.01).

The responses of 691 V1 neurons (from two monkeys), 494 V4 neurons (from two monkeys), and 294 IT neurons (from three monkeys) to the 64 images were recorded. In the present study, we compared these responses from V1, V4, and IT neurons with responses in each layer of a DCNN saliency map model. In the experiments conducted by Tamura et al. (2016), the monkeys were analgesized and paralyzed because some of the sessions required >1 h of stable recording. We cannot rule out the possibility that this procedure affected the neuronal responses. However, in previous works, the stimulus selectivity of V1 and IT neurons recorded from anesthetized/paralyzed monkeys was shown to be similar to that of awake-behaving monkeys (Wurtz, 1969; Tamura and Tanaka, 2001), which indicates that any effect of such preparation was likely to be immaterial, if it existed.

### DCNN model for the generation of a saliency map

To understand the mechanism of attentional selection for computer vision and the visual system, we used the DCNN saliency map model proposed by Pan et al. (2016), which uses simple feedforward networks, such as AlexNet (Krizhevsky et al., 2012) and VGG16 (Simonyan and Zisserman, 2014). The relatively simple network might be biologically suitable for understanding the mechanism of the bottom-up saliency map and attentional selection. By contrast, to achieve significantly accurate gaze prediction, other DCNN saliency map models (Kümmerer et al., 2014, 2017; Pan et al., 2017; Liu and Han, 2018) have been developed with complicated architectures. Additionally, in some models, the trained networks of AlexNet and VGG16 for object classification have been used to extract visual features. We speculate that the complicated CNN architectures of these models are distinct from the neural system involved in attentional selection.

Figure 1*A* shows the CNN architecture of the saliency map model proposed by Pan et al. (2016). This DCNN consists of nine convolutional, two max pooling, and one deconvolutional layers. “Conv (7 × 7, 96)” in layer 1 indicates that this layer has 96 convolutional filters with the spatial size (width × height) of 7 × 7 pixels. Because the input images consist of the three-color maps of RGB, the filters are actually represented as three-dimensional (3D) arrays with 7 × 7 × 3 elements. The application of such a 3D filter to an input image with 320 × 240 × 3 elements generates a 2D array, called a “feature map,” with 320 × 240 elements; in this study, we refer to each element as a “model neuron.” Note that 96 feature maps are generated in total as the output of the first convolutional layer in the DCNN proposed by Pan et al. (2016) because one corresponding feature map is generated from each filter, which indicates that the first convolution produces the 3D array *F* with 320 × 240 × 96 elements. Each feature map comprising the 3D array *F* is referred to as a “channel” of this layer. The *c*-th channel F(*,*,c) represents the existence of a specific visual feature extracted by the *c*-th convolutional filter (Krizhevsky et al., 2012; Zeiler and Fergus, 2013). The element F(x,y,c) represents the responses of a model neuron placed at the spatial location (x,y) for such a visual feature. Note that the characteristics and selectivity of each filter used for extracting a feature are autonomously determined via error backpropagation learning. Other convolutional layers represented by “Conv (W × H, C)” generate C feature maps based on C′ maps in the previous layer, where C′ corresponds to the number of channels in the previous layer. As shown in the first layer, the convolutional filters actually form 3D arrays with W × H × C′ elements. The convolutional operation using the *c*-th filter $m_{c}$ (with W × H × C′ elements) in a specific convolutional layer is defined as follows:

(1) $$F\left(x,y,c\right)=\overset{C^{\prime }}{\underset{k=1}{\sum }}\overset{w}{\underset{i=-w}{\sum }}\overset{h}{\underset{j=-h}{\sum }}m_{c}\left(i,j,k\right)F^{\prime }\left(x+i,y+j,k\right),$$

where $F^{\prime }$ represents a 3D array that includes the C′ feature maps in the previous layer. If the current layer is allocated after the input layer, then the RGB image array corresponds to $F^{\prime }$. *w* and *h* denote $\frac{W}{2}$ and $\frac{H}{2}$, respectively, where ⌊⋅⌋ represents the flooring function. Note that the number of channels is fixed after the max pooling and normalization of the layers in the DCNN. Additionally, the spatial size of the feature maps is fixed after the process in the convolutional layers because the zero-padding approach is applied to the network. In this network, model neurons in each convolutional layer are activated by a rectified linear unit (ReLU; Nair and Hinton, 2010; Krizhevsky et al., 2012) nonlinearity. The first two convolutional layers are followed by pooling layers, which decrease the width and height of the feature maps in the intermediate layers by a factor of four. After the final convolutional layer (Fig. 1*A*, layer 9), a deconvolution layer is used to generate a saliency map that corresponds to the width and height of the input images.

In this study, the filters of the DCNN used for generating the saliency map are randomly initialized. A variety of natural images with an intensity value ranging from zero to one (RGB color images, 320 × 240 pixels) are provided to the input layer of the network. In the original study (Pan et al., 2016), human behavioral data obtained by recording mouse tracking (Jiang et al., 2015) were provided to the DCNN for learning attentional characteristics. However, to train the DCNN, we apply human-fixation data to the network as the ground truth images with an intensity value ranging from zero to one (eight-bit gray-scale images, 320 × 240 pixels; Fig. 1*B*). We use various saliency datasets, including natural images and human-fixation data, as the training data (Judd et al., 2009; Borji and Itti, 2015; Bylinskii et al., 2015). To increase the number of training data, we also use mirror images with respect to the vertical midline. In total, we prepare 11,580 natural images and human-fixation data to train the network and produce the saliency map model as the output of the network.

We train the network using adaptive moment estimation (Kingma and Ba, 2014) with Euclidean loss between the output images of the network and the ground truth images (Fig. 1*B*). To apply this optimizer to the network, we set the learning rate parameter, *α*, to 5.0 × 10^−5^. Moreover, we set the batch size to 20 images per 250 epochs. Network training using ZOTAC GeForce GTX 1080 Ti GPU running the Chainer framework (version 1.23.0) requires ∼170 h (Tokui et al., 2015). We repeat the training of the network for 10 trials and obtain 10 distinct trained DCNN saliency map models to validate our analyses. We refer to the DCNN saliency map model based on the training of 250 epochs as the trained model. The code for a saliency map model based on DCNN is available as Extended Data 1.

10.1523/ENEURO.0200-20.2020.ed1 Extended Data 1 Codes for training the DCNN saliency map model (Pan et al., 2016). We used the Chainer framework (version 1.23.0) for this study. This code does not run under the latest version of Chainer. Download Extended Data 1, ZIP file

We perform simulations of the trained DCNN model via the 64 images (Fig. 2) used in Tamura et al. (2016). To apply the stimulus set to the trained DCNN model, we physically place these images with the original dimensions (256 × 256 pixels) with RGB values in front of a gray background image (320 × 240 pixels; intensity value of 0.5), that is, we remove regions of eight pixels from the top and bottom of the images of natural object surfaces. We record the activities of all model neurons in each layer of the trained DCNN model with respect to each of the natural object surface images, which we use for comparing the characteristics of the neural representation on visual cortices.

### Data analysis

#### Representational dissimilarity matrices (RDMs)

Kriegeskorte et al. (2008) demonstrated that RDMs allow the direct comparison of neural representations between a monkey IT and human IT, although they used radically different measurement modalities for these two species (single-cell recording for monkeys and functional resonance imaging for humans). We used RDMs to compare the characteristics of the responses in the DCNN saliency map model with those of the neural representation in V1, V4, and IT.

We computed the representational dissimilarity (*RD*) between all pairs of natural object surfaces (Kriegeskorte et al., 2008; Hiramatsu et al., 2011; Goda et al., 2014) based on the firing rates of V1, V4, and IT neurons recorded by Tamura et al. (2016). To compute the RDMs, we standardized the mean firing rates based on the Gaussian distribution with a mean of zero and a variance of one with respect to each neuron in the visual cortices. We computed the representational dissimilarity *RD_v_* between two natural object surfaces (*#i* and *j*) with respect to the rates of V1, V4, and IT neurons based on the correlation distance as follows:

(2) $$RD_{v}(i,j)=1-\frac{\sum_{n}\left(f_{n,i}^{v}-\bar{f_{i}^{v}}\right)\left(f_{n,j}^{v}-\bar{f_{j}^{v}}\right)}{\sqrt{\sum_{n}{\left(f_{n,i}^{v}-\bar{f_{i}^{v}}\right)}^{2}}\sqrt{\sum_{n}{\left(f_{n,j}^{v}-\bar{f_{j}^{v}}\right)}^{2}}},$$

where *v* represents the visual cortices (V1, V4, or IT); *i* and *j* represent the natural object surface number (1≤i,j≤64); *n* is the identity of the neuron; $f_{n,i}^{v}$ represents the firing rates of the neuron *n* in the visual cortex *v* when the object surface *#i* is presented; and $\bar{f_{i}^{v}}$ represents the mean rates of the neural population of *v* to the object surface *#i*. We computed the representational dissimilarity *RD_v_(i, j)* across the population of biological neurons in the monkeys (Kiani et al., 2007; Haxby et al., 2011). The *RD_v_(i, j)* exhibited an intensity value ranging from zero to two. If the neuronal response patterns for natural object surfaces *i* and *j* were identical, the intensity of the *RD_v_(i, j)* became zero. By contrast, the *RD_v_(i, j)* increased as the level of representational dissimilarity between response patterns for two stimuli increased. We computed the *RD_v_(i, j)* with respect to all 2016 pairs of natural object surfaces, which were summarized and represented as percentiles for each element of the RDMs (Kriegeskorte et al., 2008). Each element of the RDMs represented the comparison of the response patterns across neurons induced by two stimuli. Note that each RDM was symmetric, with a diagonal of zeros.

In the same manner, we computed the representational dissimilarity *RD_l_* between all input image pairs based on the activities of model neurons in the layer of the DCNN saliency map model as follows:

(3) $$RD_{l}(i,j)=1-\frac{\sum_{n}\left(a_{n,i}^{l}-\bar{a_{i}^{l}}\right)\left(a_{n,j}^{l}-\bar{a_{j}^{l}}\right)}{\sqrt{\sum_{n}{\left(a_{n,i}^{l}-\bar{a_{i}^{l}}\right)}^{2}}\sqrt{\sum_{n}{\left(a_{n,j}^{l}-\bar{a_{j}^{l}}\right)}^{2}}},$$

where *l* represents the layers in the DCNN saliency map model (Fig. 1*A*); $a_{n,i}^{l}$ represents the activities of model neuron *n* in layer *l* of the DCNN model with respect to the object surface *i*; and $\bar{a_{i}^{l}}$ represents the mean activities of the model neuron population of layer *l* to the object surface *i*. Note that we used all model neurons from all channels of each layer in the DCNN model to compute *RD_l_(i, j)*. We summarized *RD_v_(i, j)* as shown in Equation 2.

We used Pearson’s correlation coefficient to quantify the correspondence between the RDMs for the monkey V1, V4, and IT and those for each layer of the DCNN saliency map model. The correspondence *r_vl_* between visual cortices and the DCNN saliency map model is defined as follows:

(4) $$r_{vl}=\frac{\sum_{i=1}^{63}\sum_{j=i+1}^{64}\left(RD_{v}(i,j)-\bar{RD_{v}}\right)\left(RD_{l}(i,j)-\bar{RD_{l}}\right)}{\sqrt{\sum_{i=1}^{63}\sum_{j=i+1}^{64}{\left(RD_{v}(i,j)-\bar{RD_{v}}\right)}^{2}\times }\sqrt{\sum_{i=1}^{63}\sum_{j=i+1}^{64}{\left(RD_{l}(i,j)-\bar{RD_{l}}\right)}^{2}}},$$

where *v* and *l* represent the visual cortex (V1, V4, or IT) and the layer in the DCNN saliency map model (Fig. 1*A*), respectively. We computed *r_vl_* using 2016 RDM elements representing response patterns with respect to distinct pairs of natural object surfaces. $\bar{RD}$ represents the mean intensity of these 2016 RDM elements. Because the intensity of the diagonal elements of the RDM [*RD(i, i)*] became zero, we removed these diagonal elements from our analysis.

#### Partial correlation analyses between monkey visual areas and the DCNN saliency map model

To understand the characteristics of the responses in the DCNN saliency map model in greater detail, we computed the partial correlation of RDMs between the specific visual cortex and each layer of the DCNN saliency map model, which removed the effects of other visual cortices. The partial correlation is defined as follows:

(5) $$r_{lx\cdot y}=\frac{r_{lx}-r_{xy}\cdot r_{ly}}{\sqrt{1-r_{xy}^{2}}\sqrt{1-r_{ly}^{2}}},$$

where *r_lx·y_* is the magnitude of the partial correlation between the activities of model neurons from the specific *l*-th DCNN layer (layer *l*) and the neuronal firing rates of visual cortex *x* required for removing the effect of visual cortex *y*; and *r_lx_*, *r_xy_*, and *r_ly_* are the correlation of RDMs between the activities of DCNN model neurons in layer *l* and the rates of visual cortex *x*, between visual cortices *x* and *y*, and between model neurons in layer *l* and visual cortex *y*, respectively.

## Results

**Figure 3.**
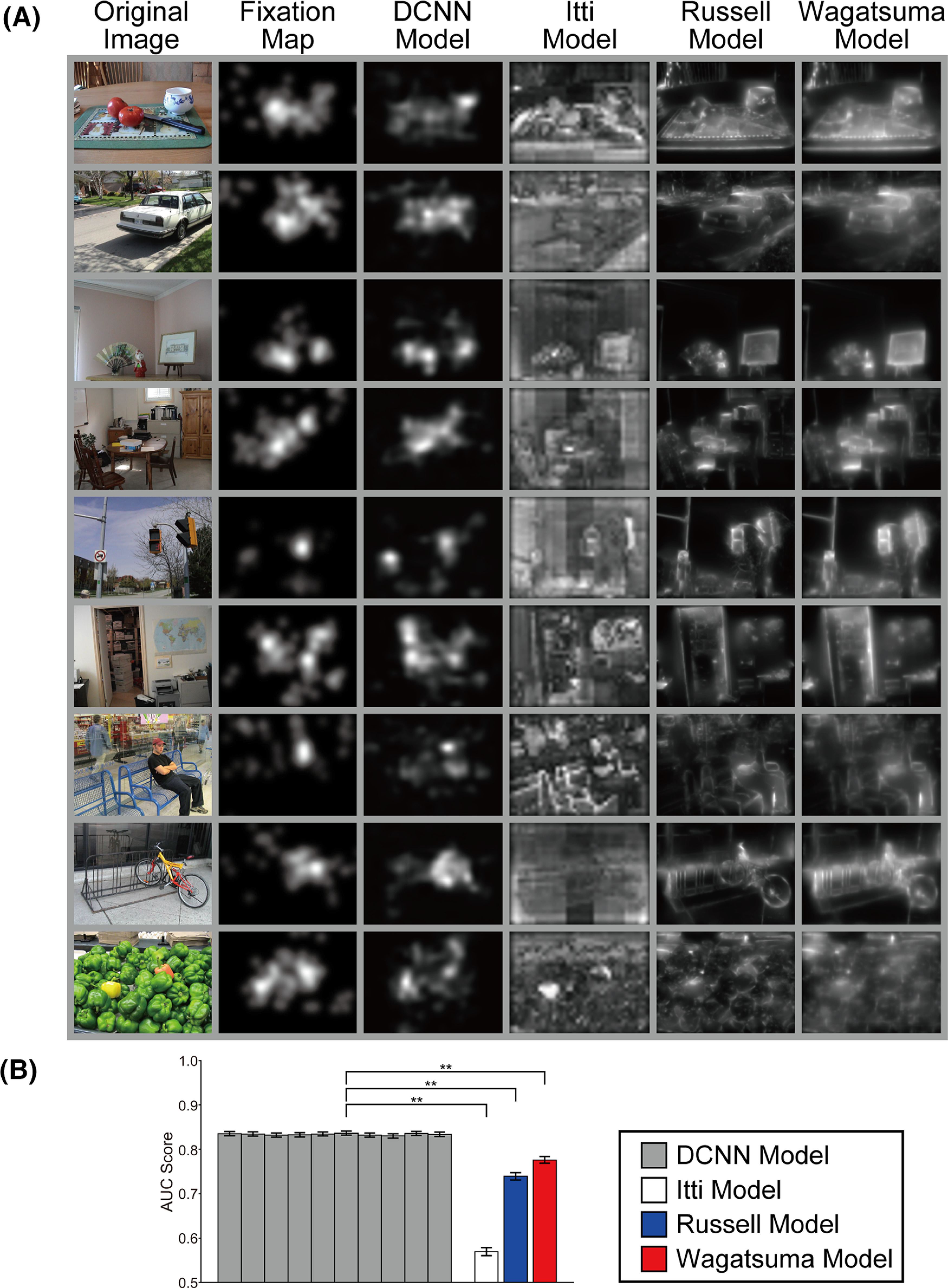
Responses of the trained DCNN saliency map models proposed by Pan et al. (2016) and previous models based on biologically plausible mechanisms (Itti and Koch, 2000; Russell et al., 2014; Wagatsuma, 2019). ***A***, Example images from the Toronto dataset (first column; Bruce and Tsotsos, 2009), associated eye-fixation maps (second column), and saliency maps calculated using the trained DCNN model (third column), Itti model (fourth column), Russell model (fifth column), and Wagatsuma model (sixth column). ***B***, Mean AUC scores of trained DCNN models and previously proposed models with respect to the Toronto dataset (120 natural images). In this work, we obtained 10 DCNN saliency map models that were independently trained with distinct initialization states and using a random order of image batches. There was no significant difference in the AUC scores among these 10 trained models (ANOVA, *p *=* *0.997). Error bars represent SEM. Asterisks indicate a significant difference in AUC scores between models (**p <* *0.01 by *t* test).

We first investigated whether the trained DCNN models reproduced the characteristics of human attentional selection for natural images. Examples of data from the Toronto dataset (Bruce and Tsotsos, 2009), associated eye-fixation maps, and a saliency map calculated using the trained DCNN (Pan et al., 2016), Itti (Itti and Koch, 2000), Russell (Russell et al., 2014), and Wagatsuma models (Wagatsuma, 2019) are shown in Figure 3*A*. The responses of the DCNN saliency map model were sparsely distributed and qualitatively similar to the characteristics of the human eye-fixation maps. To validate the mechanism underlying the trained DCNN model, we obtained 10 DCNN saliency map models that we trained independently with distinct initialization states, with the order of the image batches randomized. The prediction accuracy indices based on a receiver operating characteristic (ROC) curve analysis (Green and Swets, 1966) of the 10 trained models on the Toronto dataset are shown in Figure 3*B*. We computed the mean score of the area under the curve (AUC) for the ROC curve with respect to all 120 images in the dataset (see also Wagatsuma, 2019). There was no significant difference in the AUC scores among the 10 trained models (ANOVA, *p *=* *0.997). The AUC score of the 10 trained models was significantly higher than that of the Itti model (*t* test, *p *<* *0.01), Russell model (*t* test, *p *<* *0.01), and Wagatsuma model (*t* test, *p *<* *0.01). Note that the gaze prediction accuracy including the AUC score of recent DCNN saliency map models (Kümmerer et al., 2017; Pan et al., 2017; Liu and Han, 2018) was better than that of Pan et al.'s (2016) model (also see Pan et al., 2017, their Table 5). However, the architectures of these state-of-the-art DCNN models are complicated and distinct from biological visual systems for the bottom-up saliency map and attentional selection.

### Correspondence based on RDMs between monkey visual cortices and layers of the DCNN saliency map model

**Figure 4.**
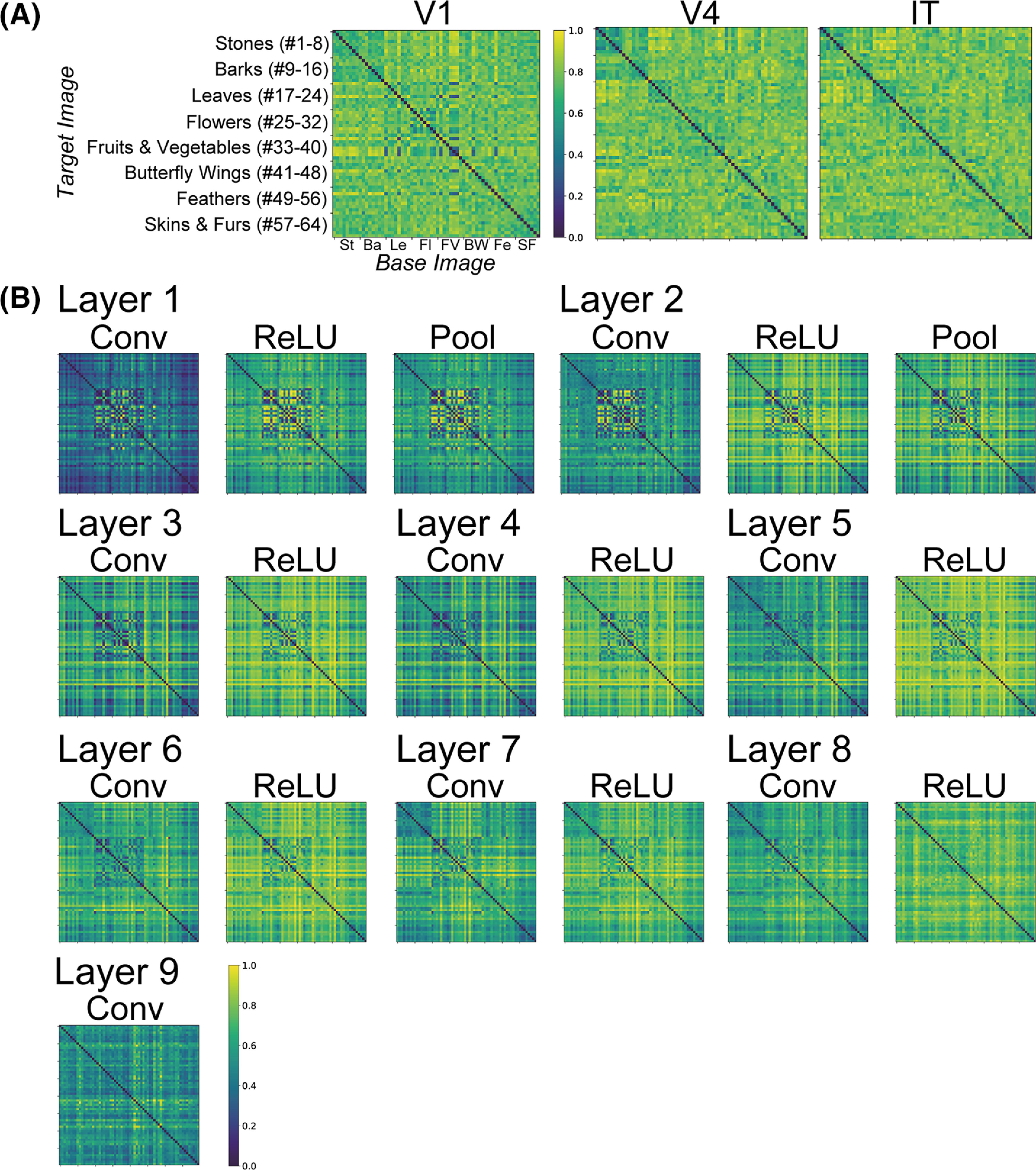
RDMs (Kriegeskorte et al., 2008; Hiramatsu et al., 2011; Goda et al., 2014) based on the responses to natural object surfaces (see also Fig. 2). Each element of an RDM represents the comparison of the response patterns induced by two natural object surfaces. We normalized the intensities of RDM elements ranging between zero and one. The intensity of the RDM element increased with the increase in the level of the representational dissimilarity between two response patterns (see also Eq. 2). ***A***, RDMs based on the responses of monkey visual cortices, V1, V4, and IT. We computed the representational dissimilarity between all pairs of stimulus images based on the firing rates of V1, V4, and IT neurons (Tamura et al., 2016). ***B***, Mean RDMs based on the activities of model neurons from the DCNN saliency map models, which are shown from the activities of 10 trained models. To compute the RDMs, we used the activities of all model neurons in all channels of each layer of the DCNN model as the neural population activities.

Figure 4*A*,*B* show RDMs based on the neural representation of monkey visual areas and activities in model neurons in the layers of the trained DCNN saliency map model, respectively. Each element of a given RDM compares the response patterns induced by two natural object surfaces (Fig. 2; Tamura et al., 2016; see also Materials and Methods).

**Figure 5.**
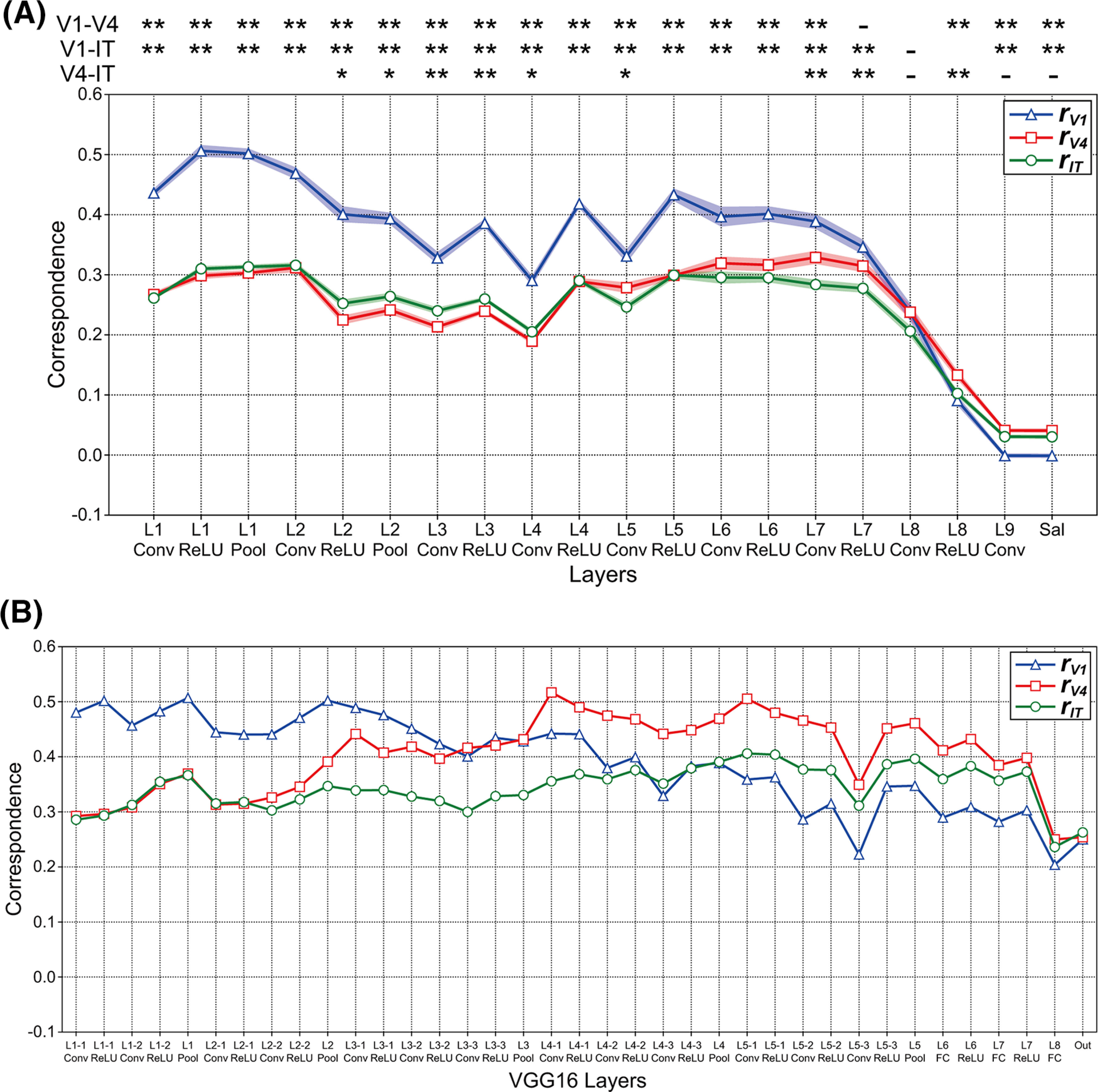
Correspondence *r_vl_* between the responses of DCNN models and the neural representation in the three visual cortices (V1, V4, and IT). ***A***, Magnitude of the correspondence *r_vl_* between the three visual cortices (V1, V4, and IT) and each layer of the DCNN saliency map model averaged over the 10 models. To examine the correspondence between the DCNN saliency map model and monkey visual cortices for representations of natural object surfaces, we computed the correlation coefficient *r_vl_* between the RDMs for the responses in each layer of the DCNN saliency map model and that for the neural representation in visual cortices V1, V4, and IT. We obtained the models via the training of 250 epochs (trained model). The *x-axis* indicates the layer of the DCNN saliency map model (see also Fig. 1*A*). The blue, red, and green lines indicate the correspondence for V1 (*r_V1_*), V4 (*r_V4_*), and IT (*r_IT_*), respectively. Shaded areas represent SEM for 10 trained models. Asterisks indicate a significant difference in the magnitudes of correspondence *r_vl_* between two visual cortices (*t* test: **p <* *0.01, *p <* *0.05, -*p *<* *0.1). The correspondence for V1 with a latency of 40 ms (Extended Data Fig. 5-1) exhibited characteristics similar to that with a latency of 80 ms (blue line). Correspondences between the three visual cortices and 10 distinct trained DCNN saliency map models are summarized in Extended Data Figure 5-2. ***B***, Magnitude of the correspondence *r_vl_* between each layer of the trained VGG16 model provided by MATLAB (MathWorks) and the three visual cortices. The conventions were the same as those used in ***A***. We observed similar patterns via the analysis of the trained VGG16 model provided by the Chainer framework (version 1.23.0; Extended Data Fig. 5-3).

We computed the correlation coefficient *r_vl_* between RDMs for the neuronal firing rates in V1, V4, and IT (Fig. 4*A*) and that for the activities in model neurons of each layer of the DCNN saliency map model (Fig. 4*B*). The correspondence *r_vl_* is defined in Equation 4. We hypothesize that the RDMs for the visual cortex are markedly correlated with that of the layer of the DCNN saliency map model if the characteristics of the responses in the model layer are similar to the neural representation in the monkey visual cortex. Figure 5*A* summarizes the magnitude of the correspondence *r_vl_* between the three visual cortices and each layer of the DCNN saliency map model averaged over the 10 trained models. The blue, red, and green lines represent the correspondence for V1, V4, and IT, respectively. For almost all levels of the DCNN layer (from layer 1 to 7), the magnitudes of the correspondence *r_V1_* based on the rates of V1 (Fig. 5*A*, blue line) were consistently higher than those based on the rates of other cortices, which implies that the characteristics of responses in the DCNN saliency map model are in agreement with that of the neural representation in V1. By contrast, the correspondence based on V4 (*r_V4_*, red line) indicates similar magnitudes and modulation patterns to that based on IT (*r_IT_*, green line). Regardless of the type of visual cortex, we found two marked peaks of correspondence *r_vl_* at early (layers 1 and 2) and higher-intermediate (layers 5 and 6) layers of the saliency map model based on the DCNN. These results suggest that the neural representations in V1 play an important role in computing the visual saliency that mediates attentional selection and in determining human gaze location.

10.1523/ENEURO.0200-20.2020.f5-1 Extended Data Figure 5-1 Correlation coefficients *r_V1_* between the firing rates in V1 with a response latency of 40 ms and the activities in model neurons of each layer of the DCNN saliency map model averaged over 10 trained models. The conventions are the same as those in Figure 5*A*. The correspondence based on V1 with a latency of 40 ms indicated characteristics similar to that with latency of 80 ms (Fig. 5*A*, blue line). Download Figure 5-1, TIF file.

10.1523/ENEURO.0200-20.2020.f5-2 Extended Data Figure 5-2 Correlation coefficients *r_vl_* between the three monkey visual cortices (V1, V4, and IT) and 10 trained DCNN saliency map models. We trained these DCNN saliency map models independently with distinct initialization states, and randomized the order of the image batches. From layer 1 to layer 7, the characteristics of responses in the 10 models were more coincident to V1 than the other two visual cortices. However, these correspondence magnitudes were different among the 10 models. Download Figure 5-2, TIF file.

10.1523/ENEURO.0200-20.2020.f5-3 Extended Data Figure 5-3 Magnitude of the correspondence *r_vl_* between each layer of the trained VGG16 model provided by the Chainer framework (version 1.23.0) and the three visual cortices. As shown in Figure 5*A*, the blue, red, and green lines indicate the correspondence for V1 (*r_V1_*), V4 (*r_V4_*), and IT (*r_IT_*), respectively. We observed similar results via the analysis of the trained VGG16 model provided by MATLAB (MathWorks; Fig. 5*B*). Download Figure 5-3, TIF file.

Additionally, recent physiological studies have reported figure–ground modulation in V1 neurons (Poort et al., 2012, 2016). Our results suggest a possible mechanism in which model neurons in early layers prefer the boundaries and contours of the presented images, whereas model neurons in higher-intermediate layers selectively respond to figural regions. This possibility will be discussed further in Discussion.

Recent physiological studies have reported that the visual response latency in V1 is ∼40–60 ms after stimulus onset (Poort et al., 2016; Yan et al., 2018). In our study, regardless of the level of visual cortex, the neuronal responses were compensated for by considering the response latency of 80 ms (see also Materials and Methods), which was longer than that reported by the aforementioned studies. We computed the correlation coefficient *r_V1_* between RDMs for the neuronal firing rates in V1 with a response latency of 40 ms and that for the activities in model neurons of each layer of the DCNN saliency map model averaged over the 10 trained models. The results for the correspondence for V1 with a latency of 40 ms (Extended Data Fig. 5-1) exhibited characteristics similar to that with a latency of 80 ms (Fig. 5*A*, blue line). Our results were not dependent on the window for neuronal response analysis.

We randomly initialized these DCNN saliency map models to obtain 10 distinct network models. The magnitude of correspondence *r_vl_* between the three monkey visual cortices and 10 distinct trained DCNN saliency map models are summarized in Extended Data Figure 5-2. As shown in Figure 5*A*, from layer 1 to layer 7, the characteristics of responses in the 10 models were more coincident to V1 than the other two visual cortices. However, these correspondence magnitudes were different among the 10 models. These results imply that the random initialization of the network induced DCNN saliency map models with distinct structures but similar mechanisms, although we applied the same training data to Pan et al.’s (2016) network.

From intermediate to deep layers, Pan et al.'s (2016) network consists of convolutional layers that are activated by the ReLU function. In the intermediate and higher-intermediate layers (layers 3, 4, and 5), the ReLU activation function markedly increases the magnitude of the correspondence *r_vl_* between the activities of model neurons and the rates of physiological neurons, regardless of the level of visual cortices. This modulation through the ReLU activation function is significantly observed in the case of V1. The possible mechanisms and roles of the ReLU activation function in the intermediate and higher-intermediate layers will be discussed in Discussion.

### Correspondence based on RDMs between monkey visual cortices and layers of the trained VGG16 model for object classification

Our analyses of the DCNN saliency map model imply that the neural representations in V1 have an important role in determining visual saliency. To investigate whether the mechanism of the DCNN saliency map model is distinct from that of other DCNN models, such as object classification, we applied our methods to the VGG16 model (Simonyan and Zisserman, 2014) and computed the correspondence *r_vl_* between activities of the VGG16 model and the responses of monkey visual cortices for representations of natural object surfaces. We used the trained VGG16 model provided by MATLAB (MathWorks).

The correspondence *r_vl_* between visual cortices and each layer of the trained VGG16 model is summarized in Figure 5*B*, with the same conventions as those used in Figure 5*A*. The magnitudes of correlations based on V1 (*r_V1_*, blue line) decreased as the level of VGG16 layers increased. By contrast, the magnitudes of correspondence based on V4 (*r_V4_*, red line) increased from layer 1–1 to convolutional layer 4–1. From layer 4 to layer 7 after ReLU activation, the characteristics of responses on the VGG16 model were more coincident to V4 responses (*r_V4_*) than those of other visual cortices. Additionally, from layer 5, the magnitude of the correspondence between IT neurons and the trained VGG16 model (*r_IT_*, green line) was higher than that based on V1 (*r_V1_*). The fluctuations in the correspondence based on IT (*r_IT_*) were smaller than those observed for other visual cortices. We observed similar results in the analysis of the trained VGG16 model provided by the Chainer framework (version 1.23.0; Extended Data Fig. 5-3). These results suggest that the characteristics of the activities in early layers of the VGG16 model trained for object classification were in agreement with that of the neural representation in V1, whereas the responses of model neurons from intermediate to deep layers of the VGG16 exhibited characteristics similar to the neural representation in V4, which implies that the mechanism of the trained DCNN saliency map model might be distinct from that of VGG16 model object classification.

### Partial correlation between monkey visual cortices and layers in the DCNN saliency map model

**Figure 6.**
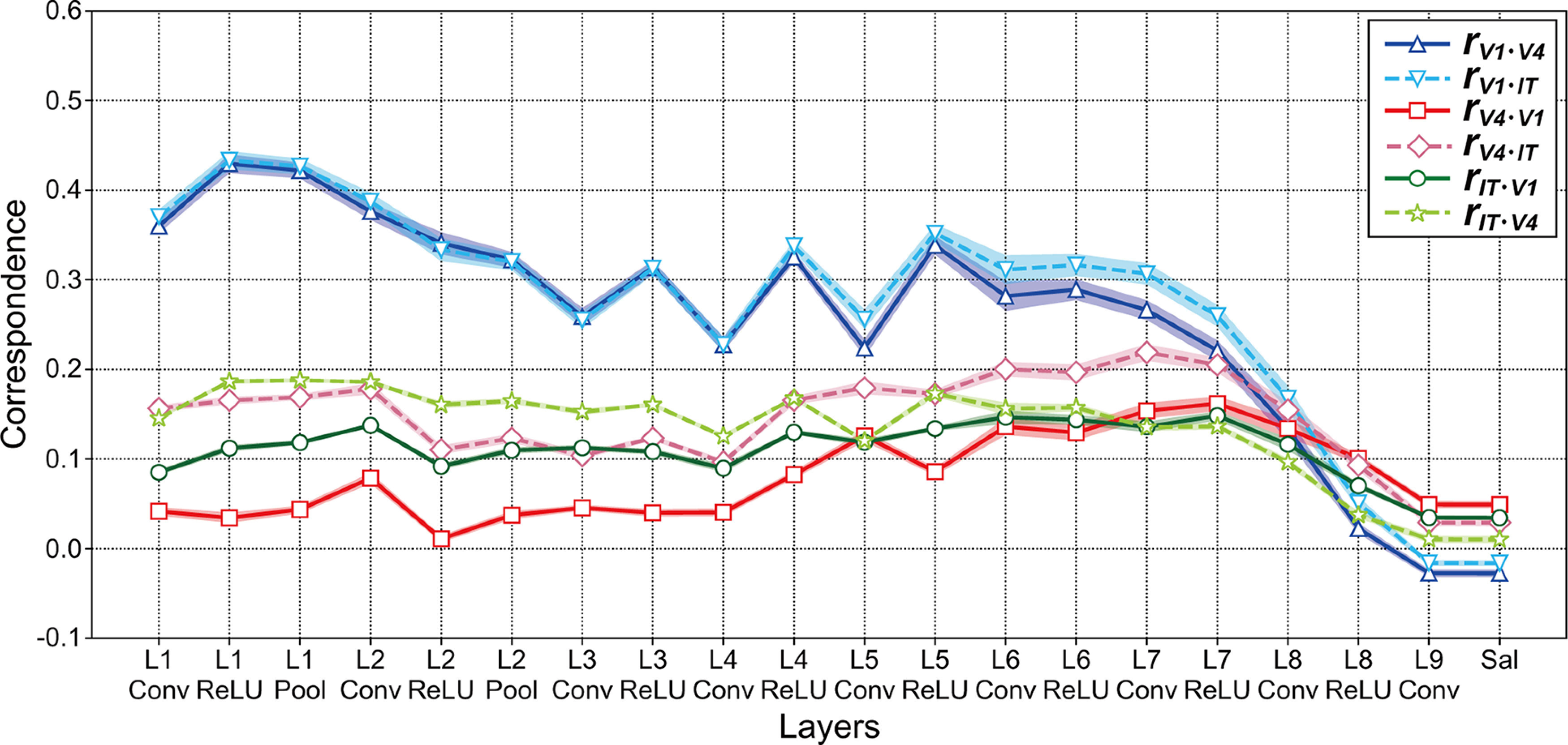
Partial correlations between each layer of the DCNN saliency map model and monkey visual cortices V1, V4, and IT. We used trained models to compute the partial correlation. We obtained data from the responses of 10 models. Shaded areas represent SEM for 10 models. The blue solid and cyan dashed lines indicate the magnitudes of the partial correlation between each layer of the DCNN saliency map models and V1 resulting from the removal of the effect of V4 and IT, respectively. The red solid and pink dashed lines indicate the partial correlation for V4 resulting from the removal of the effect of V1 and IT, respectively. Similarly, the partial correlations for IT resulting from the removal of the effect of V1 and V4 are denoted by the green solid and yellow-green dashed lines, respectively.

A partial correlation represents a correlation between two variables that results from the removal of the effects of other related variables (see also Materials and Methods; Eq. 5). The partial correlations between the DCNN saliency map model layers and monkey visual cortices are shown in Figure 6, in which the magnitudes of the partial correlation between the responses in each layer of the DCNN model and that in V1 that result from the removal of the effect of V4 and IT are represented by the blue solid and cyan dashed lines, respectively. From DCNN layer 1 to layer 6, the magnitudes of the partial correlation with V1 were markedly higher than those of the other visual cortices, as indicated by the remaining four lines. This suggests that the characteristics of the activities from early to higher-intermediate layers in the DCNN saliency map model are similar to that of the neural representation in V1. Additionally, from layer 5 of the DCNN model, the magnitudes of the V1 partial correlation resulting from the removal of the effect of V4 (blue solid line) were markedly smaller than that of IT (cyan dashed line). This result implies that, from the intermediate to deep layers of the trained model, the effect of the activities of V4 on the correspondence based on V1 responses was more significant than that of IT.

In Figure 6, the red solid and pink dashed lines represent the magnitudes of the partial correlation between each layer of the DCNN model and V4 resulting from the removal of the effect of V1 and IT, respectively. Similarly, the green solid and yellow-green dashed lines represent the magnitudes of the IT partial correlation that results from the removal of the effect of V1 and V4, respectively. From the DCNN layer 1 to layer 7, the magnitudes indicated by the red solid line were consistently smaller than those indicated by the pink dashed line. Additionally, the removal of the effects of V1 led to markedly smaller magnitudes of the partial correlation based on V4 (red solid line) than that based on IT (solid green line) from layer 1 to layer 4. These results imply that, among the early and intermediate layers of the DCNN saliency map model, the neural representation in V1 had more marked effects on the correspondence for V4 than IT.

### Responses of a single channel in each layer of the trained model for determining visual saliency

In our previous analyses, the activities in all model neurons from all channels of each layer of the trained model were used for examining the correspondence between the DCNN saliency map model and monkey visual cortices for the representation of natural object surfaces. To investigate the mechanism used by the DCNN model for computing visual saliency in greater detail, we quantitatively analyzed the activities in model neurons from a single channel of each layer of the DCNN model regarding the response to natural object surfaces. In this analysis, we computed the Pearson’s correlation coefficient between RDMs of the three monkey visual cortices and that of each channel of the DCNN saliency map model.

**Figure 7.**
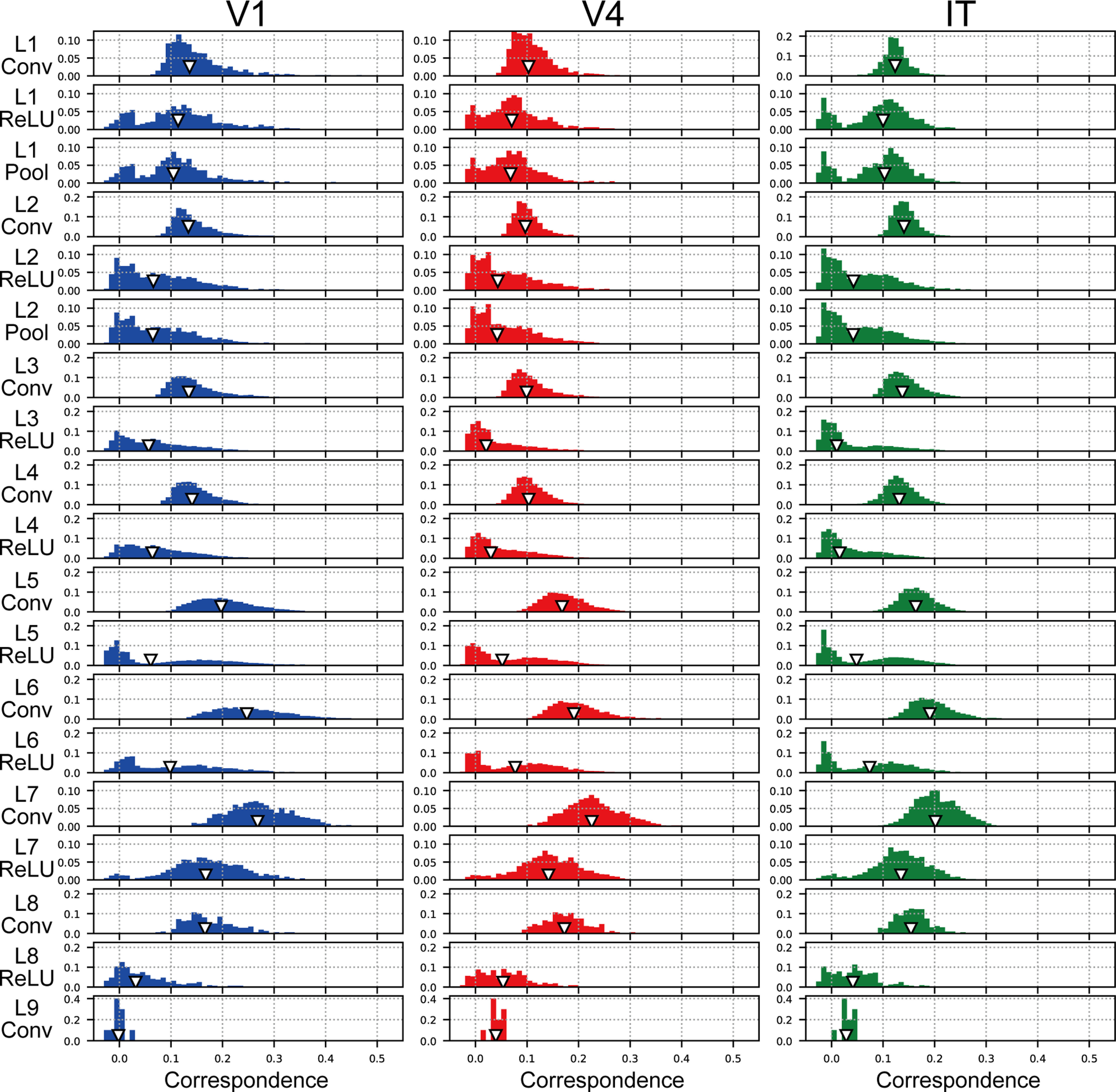
Distributions of the correspondence magnitudes between each single channel of the trained model and V1 (left column), V4 (middle column), and IT (right column). We normalized the frequency histograms of the correspondence magnitudes to the total number of channels in each layer of the 10 trained models. The triangles indicate the median values for these distributions.

The frequency histogram of the magnitudes of correspondence between each channel of the DCNN saliency map model and visual cortices is summarized in Figure 7. Regardless of the monkey visual cortex and DCNN model layer, the correspondence magnitudes displayed by most of the channels were <0.2. The distributions of the frequency histogram for convolutional layers (Fig. 7, Conv) represented a single peak. By contrast, activation via the ReLU function tended not only to induce distributions with two peaks but also consistently shifted the location of the median (Fig. 7, white triangles) toward the left, which is a pattern that was in contrast to the effects of the ReLU function identified by analyses based on the activities in all model neurons from all channels of the layer (Fig. 5*A*). These results imply that the ReLU function played a critical role in eliciting the selectivity of model neurons in DCNN saliency map model layers. This possibility will be discussed further in Discussion.

### Effects of the training epochs on the responses of the saliency map model based on the DCNN for the representation of natural object surfaces

Our analyses using RDMs implied that the responses of the trained model regarding visual saliency exhibited similar characteristics to the neural representation in V1. However, it is possible that the characteristics of the responses of the DCNN saliency map model depended on the number of training epochs. To investigate the effects of the number of training epochs on the mechanism underlying the DCNN saliency map model, we applied images of natural object surfaces to the DCNN model obtained using 10 training epochs (partially trained model) and compared the characteristics of the responses in each of its layers with those of the neural representation in V1, V4, and IT.

**Figure 8.**
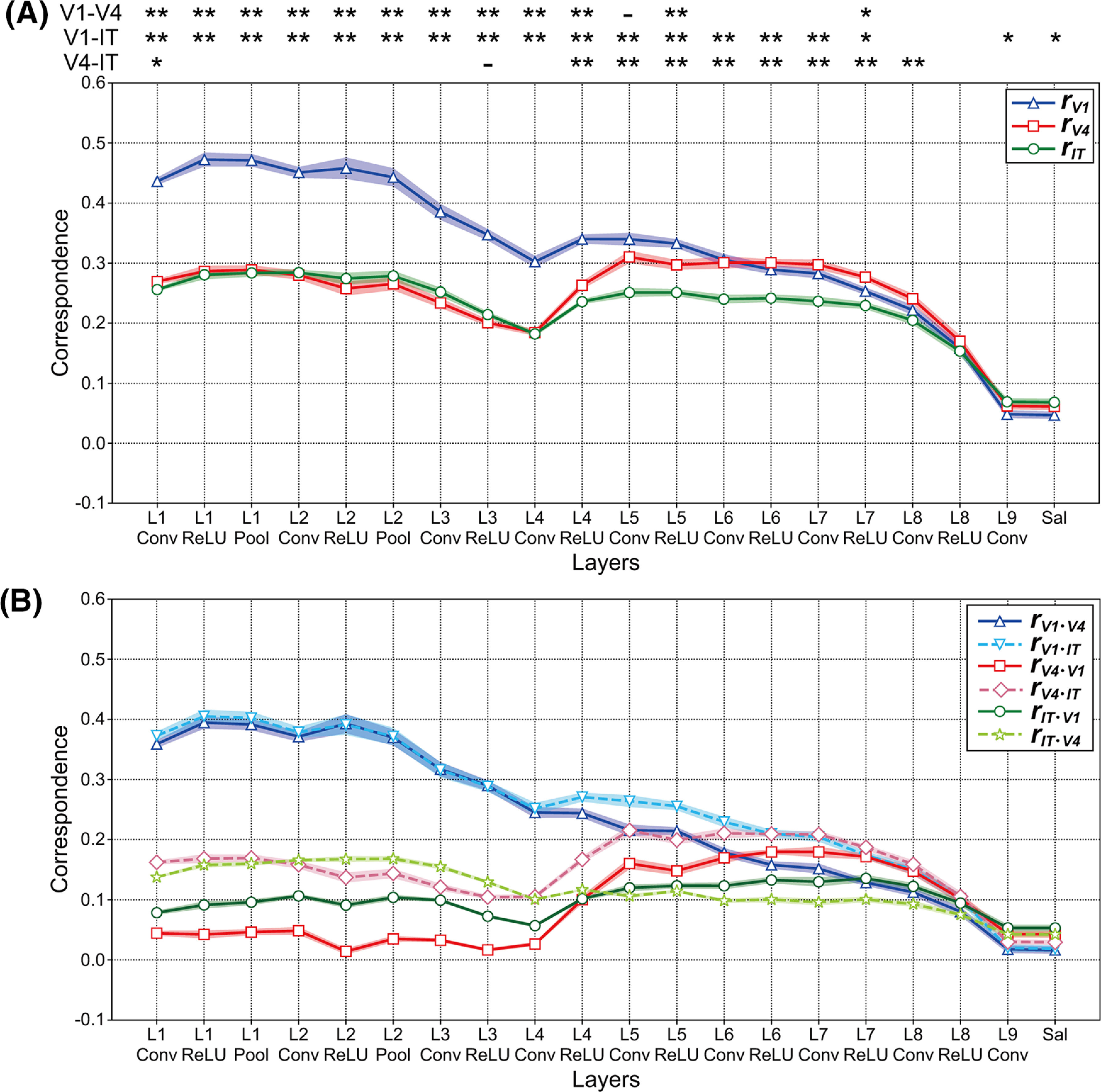
Correspondence *r_vl_* between the responses in each layer of the partially trained model (trained for 10 epochs) and the neural representation in the three visual cortices (V1, V4, and IT). We obtained the data from the responses of 10 partially trained models. The shaded areas represent SEM of 10 partially trained models. ***A***, Mean magnitudes of the correspondence *r_vl_* between the three visual cortices and each layer of the partially trained model. The conventions were the same as those of Figure 5*A*. The responses of the partially trained model in early layers seemed to be similar to the neural representations in V1. By contrast, regarding the responses in V1, the magnitudes of the correspondence *r_vl_* from layer 5 ReLU to layer 7 ReLU for the partially trained models were markedly lower than those of the trained model (also see Fig. 5*A*). Asterisks indicate a significant difference in the magnitudes of correspondence *r_vl_* between two visual cortices (*t* test: **p <* *0.01, *p <* *0.05, -*p *<* *0.1). ***B***, Partial correlations between each layer of the partially trained model and three monkey visual cortices, using the conventions described in Figure 6. The magnitudes of the partial correlations in V1 decreased as the level of DCNN layers increased. There was a marked peak in the magnitudes of the partial correlations for V4 around intermediate and higher-intermediate layers. By contrast, the magnitudes of the partial correlation for IT tended to be independent of the level of the DCNN layers.

Figure 8*A* summarizes the magnitude of the correspondence *r_vl_* between the responses in the three visual cortices and those in each layer of the partially trained model averaged over the 10 models. As shown in Figure 5*A* for the case of the trained model, the blue line represents the correspondence *r_V1_* between the partially trained model and V1. From layer 1 to layer 3, the magnitudes of the correspondence *r_V1_* between V1 and the partially trained model (Fig. 8*A*, blue line) increased to levels that were similar to those of the trained model (see also Fig. 5*A*). In particular, from layer 2 after ReLU activation to convolutional layer 3, the magnitudes of the correspondence *r_V1_* displayed by the partially trained model were markedly higher than those of the trained model. By contrast, from layer 5 after ReLU activation to layer 7 after ReLU activation, the magnitudes of the correspondence *r_V1_* of the partially trained model were markedly lower than those of the trained model. These results suggest that early layers in the DCNN saliency map model obtained a V1-like representation for natural object surfaces at early training epochs. By contrast, late training epochs might play a critical role in the development of characteristics from intermediate to deep layers in the DCNN saliency map model.

Figure 8*A*, red line, indicates the correspondence *r_V4_* between the partially trained model and V4. In contrast to the case of the correspondence *r_V1_*, there were no marked differences in the magnitude of the correspondence *r_V4_* between the partially trained (Fig. 8*A*) and trained (Fig. 5*A*) models. Additionally, from convolutional layer 6 to layer 9, the correspondence *r_V4_* for the partially trained model (Fig. 8*A*, red line) exhibited a magnitude that was similar to that of *r_V1_* for the partially trained model (Fig. 8*A*, blue line). The correspondence *r_IT_* between IT and the partially trained model is indicated in Figure 8*A*, green line. The fluctuations in the magnitudes of correspondence *r_IT_* were smaller than those observed for the other visual cortices. Additionally, in higher-intermediate layers (from layer 6 ReLU to layer 7 ReLU), the magnitudes of the correspondence *r_IT_* of the partially trained models were markedly smaller than those of the trained models (Fig. 5, green line); this finding was consistent with the characteristics of the correspondence observed for V1 (Figs. 5*A*, 8*A*, blue lines).

From layer 3 to layer 5 of the trained models, we found a marked increase in the magnitude of the correspondence after ReLU activation in all these cortical areas (Fig. 5*A*). However, in the partially trained models, the ReLU function did not markedly modulate the magnitudes of the correspondence to visual cortices, with the exception of layer 4 (Fig. 8*A*). The increase in the correspondence observed after ReLU activation in intermediate layers was a specific characteristic of the trained DCNN saliency map model.

Finally, similar to the case of the trained model, we computed the partial correlations between the responses in each layer of the partially trained models and that in a specific visual cortex via the removal of the effect of the other visual cortices (Fig. 8*B*). Figure 8*B*, blue solid and cyan dashed lines, shows the partial correlations between the partially trained model and V1 after the removal of the effect of V4 and IT, respectively. These partial correlations with V1 observed from layer 1 to layer 2 indicate magnitude levels larger than 0.3. This result suggests that the characteristics of the responses in early layers of the partially trained models corresponded to that of the neural representation in V1. Additionally, the magnitudes of partial correlations between V1 and the partially trained model decreased as the level of DCNN layers increased. From layer 5, there were similar levels of magnitude of the partial correlations among the three visual cortices, which implied that the characteristics of the responses in higher-intermediate and deep layers of the partially trained models might be distinct from those of the responses in V1, V4, and IT. These results suggest that the characteristics of the responses in intermediate layers of the DCNN saliency map model develop during late training epochs.

## Discussion

To understand the interconnections between the mechanism of the saliency map model based on DCNNs and the neural system for determining gaze location and attentional selection, we investigated the correspondence between the DCNN saliency map model (Pan et al., 2016; Fig. 1*A*) and monkey visual cortices V1, V4, and IT (Tamura et al., 2016) for representations of natural object surfaces (Fig. 2). From layer 1 to layer 7, the magnitudes of the correspondence between the activities in model neurons of trained DCNN saliency map models and the responses in V1 neurons were consistently higher than those observed for the remaining two visual cortices (Fig. 5*A*), which seemed to be distinct from the characteristics on the mechanism of the trained VGG16 model (Simonyan and Zisserman, 2014) for object classification (Fig. 5*B*). This result suggests that the activities in the trained DCNN saliency map model had similar characteristics regarding the responses to the neural representation in V1. Furthermore, our analyses implied that early layers of the DCNN saliency map model obtained a V1-like representation at early training epochs, whereas late training epochs might play critical roles in the development of the characteristics of intermediate, higher-intermediate, and deep layers (Fig. 8). These results not only provide important insight into the mechanism of the trained DCNN saliency map model, but also support the V1 saliency hypothesis (Li, 1999a, 2002; Koene and Zhaoping, 2007; Jingling and Zhaoping, 2008; Zhang et al., 2012; Zhaoping, 2014; Zhaoping and Zhe, 2015) that the neural representations in V1 play an important role in the computation of the visual saliency that mediates attentional selection.

### Comparison of physiological data during passive viewing with the DCNN saliency map model based on human-fixation data

We used the neural data from V1, V4, and IT recorded during passive viewing of natural object surfaces (Fig. 2; Tamura et al., 2016) with a presentation duration of 200 ms. These neural responses appeared to be distinct from the neural representation of a significant salient location. Additionally, in this physiological study, the eye movement of monkeys during recording was prevented using muscle relaxant (see Materials and Methods). However, the DCNN saliency map model trained based on human-fixation data indicated characteristics similar to the neural responses of V1 in a passive viewing task. These results provided evidence that neural responses in V1 play an important role for determining the salient location. This possible mechanism supports the V1 saliency hypothesis, which suggests that the neural activities in V1 underlie the neural representations for a saliency map of the visual field to exogenously guide attentional selection.

### Possible mechanisms based on the representation in V1 for determining visual saliency

The majority of neurons in V1 respond strongly to a bar stimulus presented in the receptive field of a neuron if the bar stimulus is aligned with the preferred orientation of the neuron (orientation selectivity; Hubel and Wiesel, 1968). Interestingly, early layers of the AlexNet model for object classification seem to obtain a similar profile to that of Gabor filters, which are used for modeling neurons with orientation selectivity (Itti et al., 1998; Lee et al., 1999; Deco and Lee, 2004; Sakai et al., 2012; Krizhevsky et al., 2012; Zeiler and Fergus, 2013). Additionally, the detection of the orientation from the input image is the first, and necessary, process of saliency map models based on biologically plausible mechanisms (Itti et al., 1998; Li, 1998, 1999c; Itti and Koch, 2000; Russell et al., 2014; Wagatsuma, 2019). Model neurons with orientation selectivity in early vision may play a critical role in understanding the visual scene and in computing the visual saliency that mediates attentional selection. These results imply that orientation selectivity is developed in model neurons in early layers of the DCNN saliency map model (Pan et al., 2016) for the representation of the most salient location in the visual images.

The conclusion that the neural representations in V1 play an important role in computing the salient location that mediates attentional selection and in determining human gaze is plausible based on our analyses of the DCNN saliency map model. This possible mechanism agrees with the V1 saliency hypothesis. In this hypothesis, intracortical interactions within V1 induce the contextual modulation (Allman et al., 1985; Knierim and van Essen, 1992; Li and Li, 1994; Jones et al., 2001, 2002; Ozeki et al., 2009) that is necessary for emphasizing the neural representation of the unique feature in the retinal image and for homogeneously suppressing other features that represent the background (iso-feature suppression; Zhaoping and Zhe, 2015), which plays an essential role in the generation of the neural representation of the saliency map. It is possible that the connections between early and intermediate layers in the trained DCNN saliency map model (Pan et al., 2016) occur via a mechanism that is similar to the intracortical interactions within V1 that promote iso-feature suppression.

Physiologic studies have reported that the responses of neurons in V1 and V2 underlie figure–ground segregation (Zhou et al., 2000; Qiu et al., 2007; Poort et al., 2012, 2016; Martin and von der Heydt, 2015). The segregation of images into figure and background is a fundamental process in visual perception. Poort et al. (2012, 2016) performed neurophysiological experiments that indicated that the neural responses in V1 for representing the figure occurred according to the process of edge detection. Furthermore, biologically plausible saliency map models have implied that the neural mechanism of figure–ground segregation plays an important role in predicting the locations of attentional selection and in improving the prediction accuracy of the human gaze (Li, 1999a; Zhaoping, 2003, 2014; Russell et al., 2014; Wagatsuma, 2019; Uejima et al., 2020). Our analyses demonstrated that the responses of intermediate and higher-intermediate layers (layer 4 ReLU, layer 5 ReLU, and layer 6; see Fig. 5*A*) of the trained DCNN saliency map model exhibited characteristics similar to the neural representations in V1. The selective response of model neurons in these layers to the figural regions before the computation of the salient location in the input image is a possible mechanism of the DCNN saliency map model. Further analyses based on the neuronal responses to figure–ground segregation are necessary to understand the detailed mechanism underlying the DCNN saliency map model.

In contrast to the mechanisms discussed above, it is possible that only the higher-intermediate layers (from layer 5 to layer 6) of the trained DCNN saliency map model might reflect the V1 responses. A circular symmetric receptive field with a mutually antagonistic center and surround is characteristic of retinal ganglion cells and neurons in the lateral geniculate nucleus (LGN; Rolls and Deco, 2002), which generate the inputs to biological V1 neurons and are modeled using the difference of Gaussian (Russell et al., 2014). It is plausible that model neurons in early layers of the DCNN saliency map model may include characteristics similar to retinal ganglion cells and LGN neurons to allow them to produce V1-like model neurons in higher-intermediate layers. Neural responses in the retina and LGN might be informative for further understanding the mechanism of the DCNN saliency map model.

In our analyses, responses in deep layers (from layer 8 to output layer of the DCNN model (Sal)) of the trained DCNN saliency map models indicated distinct characteristics from the neural representations in the three visual cortices (Fig. 5*A*). Particularly, in the Sal layer corresponding to the output of the DCNN saliency map model, the magnitude of the correspondence *r_V1_* based on the neural responses of V1 was the lowest among the three visual cortices. These results implied that, whereas the neural representations in V1 play an important role in computing salient locations, the neural representation of visual saliency might be represented in the brain area involved in eye movement or the visual cortex except for V1, V4, and IT. A previous study implied that the superior colliculus receives V1 responses, which plays a critical role in guiding saccades (Zhaoping, 2014). A possible mechanism suggested by our analyses is that the Sal layer exhibits similar characteristics to the neural representation in the superior colliculus. Additionally, it is possible that the most activated model neurons in early and higher-intermediate layers correspond to the activities in the Sal layer. Further analyses of correspondences between DCNN layers are necessary to understand the detailed mechanism of the neural representation of visual saliency.

### Effects of training epochs on the development of the DCNN saliency map model and on the computation of the visual saliency that mediates attentional selection

Our results showed that the characteristics of V1-like representations in early layers of the DCNN saliency map model were obtained during early training epochs, whereas the late training epochs seemed to play an important role in the development of the mechanisms from intermediate layers of the DCNN saliency map model (Fig. 8). If model neurons in intermediate and higher-intermediate layers are selective to the figural region for computing visual saliency as discussed in the previous section, the selectivity of figure–ground segregation in these layers might develop after the early layers obtain orientation selectivity and the function of edge detection, similar to V1 neurons. These results suggest that feedforward processing based on edge detection in early vision underlies the selectivity of figure–ground segregation in intermediate-level visual areas, and that rapid feedback signals from higher-level visual areas play crucial roles in the neural representation of the figural region, which might correspond to suggestions from the computational models for understanding the neural mechanism underlying figure–ground segregation (Li, 1999a; Zhaoping, 2003, 2014; Sakai and Nishimura, 2006; Craft et al., 2007; Mihalas et al., 2011; Sakai et al., 2012; Wagatsuma et al., 2016; Hu and Niebur, 2017).

### Roles of the ReLU activation function in the DCNN saliency map model

Our analysis showed that the ReLU activation function markedly increased the magnitude of the correspondence between model neurons in the intermediate layers of the trained DCNN saliency map model (layers 3, 4, and 5) and monkey visual cortices (Fig. 5*A*). The facilitation of the selectivity and sparseness of model neurons in specific layers of the DCNN model by the ReLU function is a possible mechanism for increasing this correspondence. The ReLU function is a nonlinear activation function that selects the maximum value between zero and the response of the model neuron (Nair and Hinton, 2010; Krizhevsky et al., 2012), which might function in the selection of model neurons and in the facilitation of the sparseness within the channel for representing informative characteristics of attentional selection. Previous studies aimed at understanding the neural mechanisms of sensory processing have implied that biological neurons encode sensory information based on a small number of active neurons at any given point in time (sparse coding; Olshausen and Field, 1996, 2004). If activation via the ReLU function plays an important role in the selection of a small number of active model neurons for computing visual saliency, sparse coding in the DCNN saliency map model may be reproduced by the ReLU activation function.

The responses of biological neurons in visual cortices are suppressed when stimuli with their preferred feature (iso-feature) are provided around their receptive field (Allman et al., 1985; Knierim and van Essen, 1992; Li and Li, 1994; Jones et al., 2001, 2002; Ozeki et al., 2009). Another possible role of ReLU activation in the DCNN saliency map model is the implementation of iso-feature suppression, as reported in visual cortices. In V1, inter-receptive field suppression is mainly mediated by long-distance horizontal connections from excitatory to inhibitory neurons (Adesnik et al., 2012; Chen et al., 2017). In the DCNN saliency map model, model neurons with activity greater than zero are selected via ReLU activation, which implies that the ReLU activation function selects model neurons with similar characteristics to biological excitatory neurons. If iso-feature suppression in visual cortices is implemented via the long-horizontal connections arising from excitatory neurons, the ReLU activation function might play an important role in implementing iso-feature suppression in the DCNN saliency map model. Additionally, the V1 saliency hypothesis implies the critical role of iso-feature suppression in the determination of the saliency of the location to guide attentional selection (Li, 2002; Jingling and Zhaoping, 2008; Zhang et al., 2012; Zhaoping and Zhe, 2015). If iso-feature suppression in the DCNN saliency map model is implemented based on the ReLU function, activation through the ReLU function might play a key role in the reproduction of the neural mechanism underlying attentional selection.

Our analyses using the population activities of all model neurons from all channels of each layer indicated that the ReLU activation function markedly increased the magnitude of the correspondence between monkey visual cortices and intermediate layers of the DCNN saliency map model (layers 3, 4, and 5; Fig. 5*A*). By contrast, regardless of the level of the DCNN layer, the ReLU activation function decreased the correspondence magnitudes between the RDMs of each single channel and that of the neural responses, which were demonstrated by the shift of the peak location in the distribution of the correspondence magnitude toward the left (Fig. 7). The effects of the ReLU function on the correspondence magnitude for the population activity using all model neurons in each layer were markedly distinct from those for the responses of model neurons in each single channel. The physiological V1, V4, and IT neuronal populations recorded by Tamura et al. (2016) might include various neurons with a distinct preference and selectivity. Additionally, in this work, we used the responses of all physiological neurons to compute the RDMs (see Materials and Methods). Assuming that each channel in a layer of the DCNN saliency map model expresses a preference for a specific visual feature or selectivity to specific visual information, in Figure 7, we compare the characteristics of the neural population activities with various levels of selectivity to those of model neurons with a preference for a specific visual feature. A future study using a neuronal population with selectivity to a specific visual feature is necessary to understand the mechanism of the DCNN models in greater detail.

### Further understanding of the mechanisms for various saliency map models by applying them to the methods and metric used in this study

In this study, we used RDMs (Kriegeskorte et al., 2008) to compare the characteristics of the responses of the DCNN saliency map model with those of the neural representation in visual cortices. Our analysis methods and metrics used in this study are applicable to various other saliency map models (Itti and Koch, 2000; Kümmerer et al., 2014, 2017; Russell et al., 2014; Pan et al., 2017; Liu and Han, 2018; Wagatsuma, 2019; Uejima et al., 2020). Our analysis results and the V1 saliency hypothesis implied that the activities of model neurons with similar characteristics to V1 responses were the basis for better gaze prediction accuracy for the saliency map models. Current analysis methods and metrics might be available for estimating and evaluating the performance of various saliency map models.

In conclusion, we quantitatively analyzed the DCNN saliency map model. The responses of the trained DCNN saliency map model were in agreement with the characteristics of the neural representation in V1, which seemed to be consistent with the V1 saliency hypothesis based on physiological, psychophysical, and computational studies (Li, 1999b, 2002; Jingling and Zhaoping, 2008; Zhang et al., 2012; Zhaoping, 2014; Zhaoping and Zhe, 2015). Our results not only provided important insight into the mechanism of the trained DCNN saliency map model but also suggest the critical role of the neural representation in V1 for computing the visual saliency that mediates attentional selection and for determining human gaze location.

## Synthesis

Reviewing Editor: Li Li, New York University Shanghai Decisions are customarily a result of the Reviewing Editor and the peer reviewers coming together and discussing their recommendations until a consensus is reached. When revisions are invited, a fact-based synthesis statement explaining their decision and outlining what is needed to prepare a revision will be listed below. The following reviewer(s) agreed to reveal their identity: NONE.

The quality and clarify of the ms have improved a lot but there are still some minor concerns that should be addressed before the ms can be accepted for publication. I list the detailed comments below:

This is the second review of the revision of the paper, eN-NWR-0200-20, titled “Correspondence between monkey visual cortices and

layers of a saliency map model based on a deep convolutional neural network for representations of natural images”.

The revision has substantially improved the presentation of the paper, it also has addressed most issues raised in the previous review.

In particular, various terms are defined clearly, so it is much easier to follow the details.

This paper would be stronger with another revision effort. I list the following comments and suggestions for this purpose.

(1) line 84, “monkey V1 neurons” --- I assume the authors mean “monkey neurons", i.e., delete “V1”?

(2) line 124, the monkeys were sedated during the recording, later, the paper said “passive viewing”. Plesae make it clear whether sedated monkeys can be seen as doing “passive viewing” of the images. Are they fixating on the images, please clarify.

(3) line 174, “stimulus presentation was shifted to 80 ms ...” --- this 80 ms is a bit long compared to V1’s saliency signals seen in Yan et al 2018 (cited in this paper) in which the saliency signals were seen in 40- 60 ms from stimulus onset. Please comment on why shifting on 80 ms, rather than perhaps 40 ms? Would the results in this paper still hold if the shift is 40 ms?

(4) line 343 “model neuron m from the specific DCNN layer” --- I suspect that this is a English translation error. Do you mean “m-th model neuron", or “neural responses in the m-th layer", I think you mean the latter. Please correct it. Also, in line 345, you said “DCNN model neuron m” --- the same mistake?

(5) Line 603 “Additionally, such a duration of stimulus presentation seemed to prevent monkeys from shifting the location of gaze and attentional selection” --- this is incorrect, or perhaps another English translation error, while humans take about 200 ms from visual inputs to saccade, the visual inputs before the saccade LEAD to, or CAUSE, the saccade, rather than “preventing” the saccade! Also, even before the saccade, attention often already shifts toward the saccadic target, so one should not say that the stimulus before the saccade is preventing the attentional shift!

(6) Line 622-625. “Additionally, the detection of the orientation from the input image is the first, and necessary, process of saliency map models based on biological plausible mechanisms (Itti et al 1998, ...

Wagetsuma 2019). The citation should include Li Z. (1998) Neural Computation. 10. 903-940, 1998

Li Z. (1999) Network: Computation in Neural Systems Volume 10, Number 2, May 1999. Page 187-212.

This model by Li in late 1990s is as far as I know the most biologically based model of saliency mechanisms in V1, with orientation tuned units.

(7) Line 650-653 “Furthermore, biologically plausible saliency map models ... figure-ground ...(Russell et al 2014, Wagatsuma 2019)”. Earlier models of figure-ground processing, including the analysis between edges of the figure versus center of the figures, should be cited, it is Li Z. (1999) Can V1 mechanisms account for figure-ground and medial axis effects? or PDF version Advances in Neural Information Processing Systems 12. MIT Press, Cambridge, MA, Editors: SA Solla, TK Leen \& K-R Muller P. 136-142

or its later journal version:

Zhaoping L. V1 mechanisms and some figure-ground and border effects. In Journal of Physiology, Paris 97 (4-6) 503-515 (2003)

Or you can find it in chapter 5 of Zhaoping’s book “Understanding vision: theory, models, and data” 2014.

Also related to this issue is in Line 699 of the manuscript “These results suggest that feedforward processing based on edge detetion in early vision underlies the selectivity of figure-ground segregation”.

(8) Line 680. “excepting for” should be “except for”. Please note that there are still a lot of English errors, I am not pointing them all out here, and many sentences and expressions seem to have problems that look like English translation problems. So it is important that the authors look through the paper thoroughly to make sure that these English problems are removed, and translations follow the authors’ original content and meanings faithfully. It will be a pity if good science is a bit lost in the translation.

(9) Line 682--685. “... gradually established in the dorsal visual stream based on the significant activities of V1 neurons, which is in agreement with the V1 saliency hypothesis ... Additionally, it is possible that the most activated model neurons ... corresponds to the activities in the Sal layer.”

V1 saliency hypothesis did not say that “dorsal visual stream “ gradually establishes the saliency. Rather, it is suggested in Zhaoping 2014 that superior colliculus reads out the saliency values in V1 to guide saccades.

After all, a saliency map needs to be read out in order to guide gaze, and the read out must corresponds to the most salient location (or the highest responding V1 neuron to each location, which is the saliency map).

So Sal is the read out in DCNN, and it is perhaps more likely superior colliculus that Sal is representing, i.e.,

Sal represents the read-out of the saliency map, while layers before Sal in DCNN represent the saliency map

information before the read out.

(10) This paper is very long so I am not pointing out all the detailed problems. Given that this revision has improved the quality of this paper so substantially from the previous version, I can imagine that another such revision effort could improve the paper so much more, so I highly recommend that the authors do another round of such a revision effort, the research effort behind the results is worthy of such an effort.
