# Supplementary figures and images for: Correspondence between Monkey Visual Cortices and Layers of a Saliency Map Model Based on a Deep Convolutional Neural Network for Representations of Natural Images
Source: eNeuro. 2021 Jan 12;8(1):ENEURO.0200-20.2020. doi: 10.1523/ENEURO.0200-20.2020 (PMC7890521; doi:10.1523/ENEURO.0200-20.2020)

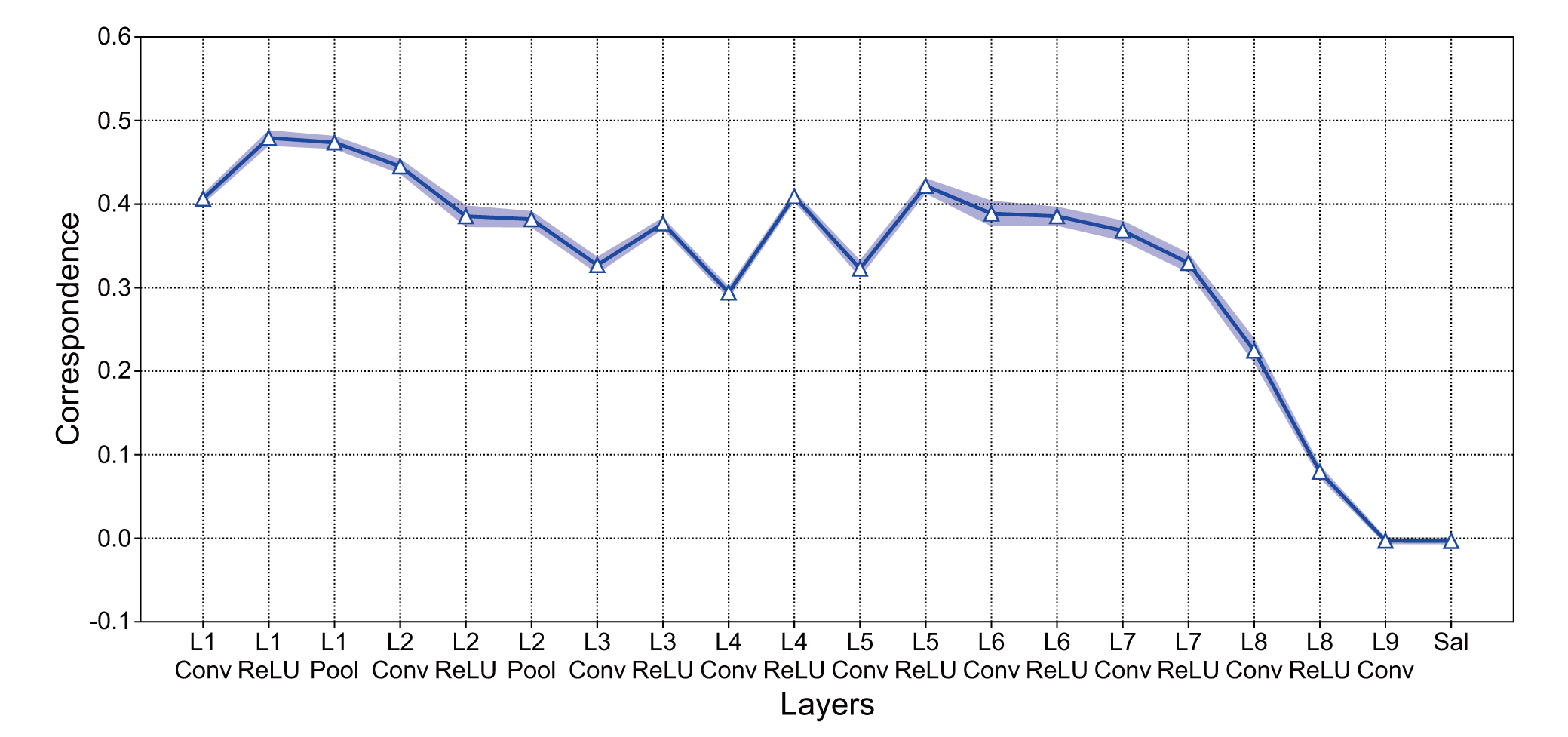

Supplement: Extended Data Figure 5-1 — Correlation coefficients rV1 between the firing rates in V1 with a response latency of 40 ms and the activities in model neurons of each layer of the DCNN saliency map model averaged over 10 trained models. The conventions are the same as those in Figure 5A. The correspondence based on V1 with a latency of 40 ms indicated characteristics similar to that with latency of 80 ms (Fig. 5A, blue line). Download Figure 5-1, TIF file. [file enu-eN-NWR-0200-20-s03.tif]

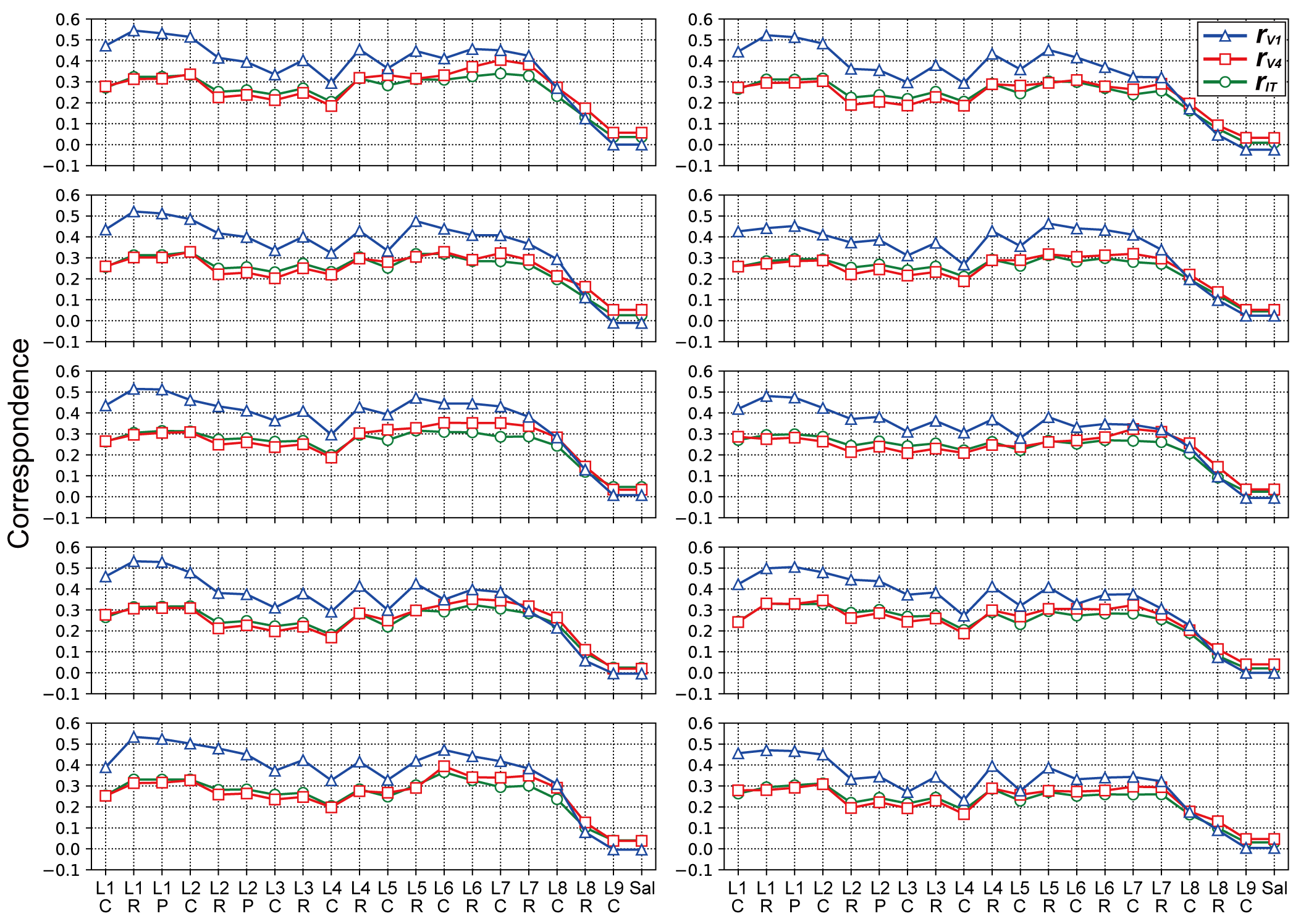

Supplement: Extended Data Figure 5-2 — Correlation coefficients rvl between the three monkey visual cortices (V1, V4, and IT) and 10 trained DCNN saliency map models. We trained these DCNN saliency map models independently with distinct initialization states, and randomized the order of the image batches. From layer 1 to layer 7, the characteristics of responses in the 10 models were more coincident to V1 than the other two visual cortices. However, these correspondence magnitudes were different among the 10 models. Download Figure 5-2, TIF file. [file enu-eN-NWR-0200-20-s04.tif]

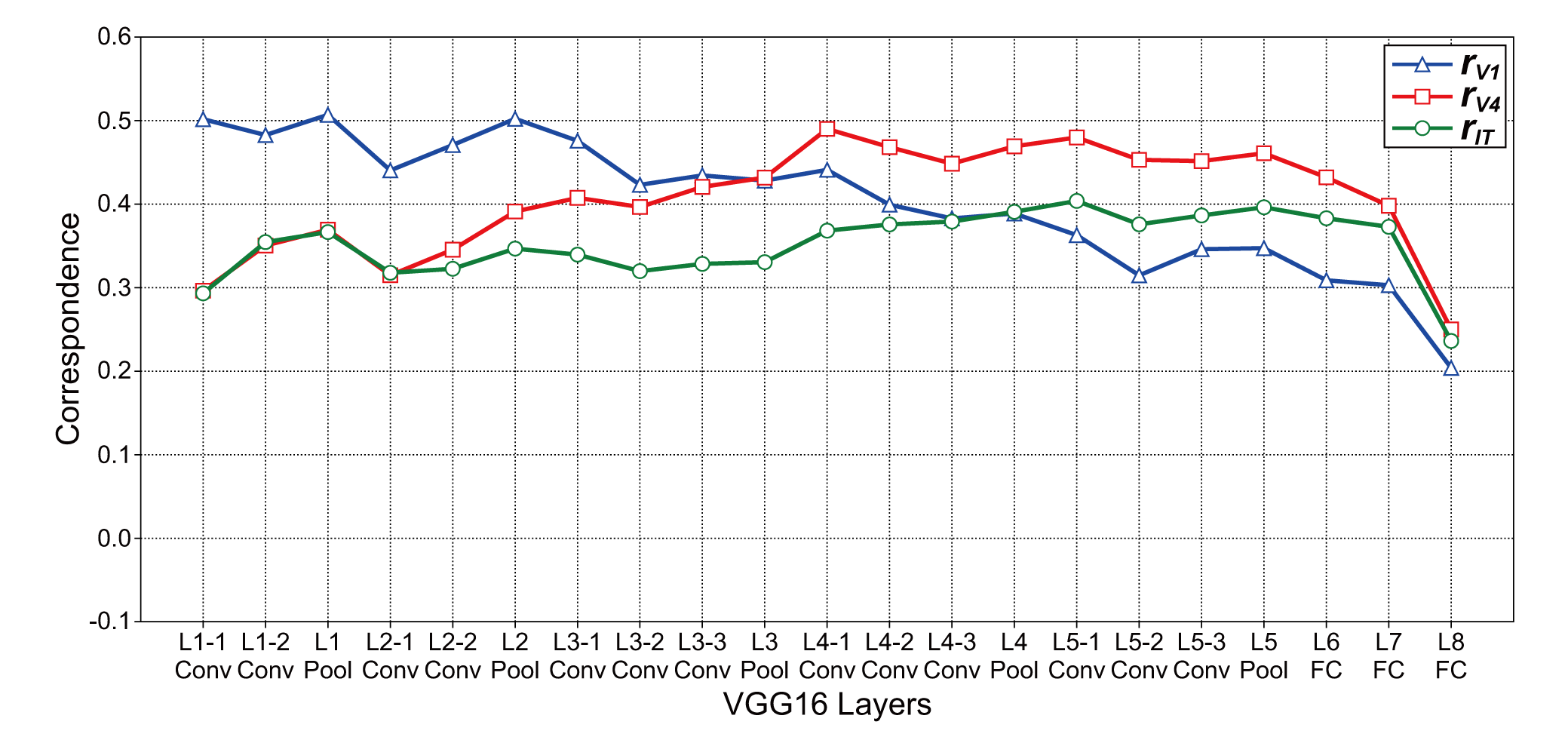

Supplement: Extended Data Figure 5-3 — Magnitude of the correspondence rvl between each layer of the trained VGG16 model provided by the Chainer framework (version 1.23.0) and the three visual cortices. As shown in Figure 5A, the blue, red, and green lines indicate the correspondence for V1 (rV1), V4 (rV4), and IT (rIT), respectively. We observed similar results via the analysis of the trained VGG16 model provided by MATLAB (MathWorks; Fig. 5B). Download Figure 5-3, TIF file. [file enu-eN-NWR-0200-20-s05.tif]
